# Socioeconomic Status and Stroke: A Review of the Latest Evidence on Inequalities and Their Drivers

**DOI:** 10.1161/STROKEAHA.124.049474

**Published:** 2024-12-19

**Authors:** Camila Pantoja-Ruiz, Rufus Akinyemi, Diego I. Lucumi-Cuesta, Daniel Youkee, Eva Emmett, Marina Soley-Bori, Wasana Kalansooriya, Charles Wolfe, Iain J. Marshall

**Affiliations:** School of Life Course and Population Sciences, King’s College London, United Kingdom (C.P.-R.).; Neuroscience and Ageing Research Unit, Institute for Advanced Medical Research and Training, College of Medicine, University of Ibadan, Nigeria (R.A.).; Escuela de Gobierno, Universidad de los Andes, Bogotá, Colombia (D.I.L.-C.).; School of Life Course and Population Health Sciences, King’s College London, United Kingdom (D.Y., E.E., M.S.-B., W.K., C.W., I.J.M.).

**Keywords:** hypertension, mortality, risk factors, social class, stroke

## Abstract

The latest research on socioeconomic status (SES) and stroke continues to demonstrate that individuals with low SES are at a higher risk of stroke, receive lower-quality care, and experience poorer outcomes. Despite growing evidence on the impact of SES on stroke, gaps remain in understanding the underlying mechanisms and the influence of SES in different contexts, particularly in low- and middle-income countries. This narrative review builds upon our previous reviews from 2006 to 2015, focusing on studies published since 2015 to update on the influence of SES on stroke. Reports from nationwide or population-based observational studies in the past decade have confirmed that these inequalities persist globally and have provided new evidence on their mechanisms. In high-income countries, inadequate control of cardiovascular risk factors (hypertension, diabetes, obesity, and dyslipidemia) among lower socioeconomic groups has been found to explain much of the inequality in stroke risk. Exposure to particulate air pollution (both environmental and indoor from solid fuel cooking) synergizes with cardiovascular risk factors, especially hypertension, as major causes in low- and middle-income countries. Lower SES is persistently associated with disparities in care and increased poststroke disability and mortality. Lower SES also exacerbates other causes of health inequality among women, ethnic minorities, and migrants. Addressing stroke inequalities requires an interdisciplinary approach. Targeting cardiovascular risk factors, providing equitable quality of acute and rehabilitative stroke care, enacting legislative measures, and implementing societal changes remain leading global priorities.

We conducted 2 literature reviews in 2006 and 2015, highlighting inequalities in stroke occurrence, severity, care, and recovery driven by socioeconomic status (SES).^1,2^ Cardiovascular risk factor (CVRF) management and stroke evidence-based care were disproportionately poorer in low- and middle-income countries (LMICs) and disadvantaged groups within high-income countries (HICs).^1,2^ Since then, new studies and extensive longitudinal analyses, alongside improved stroke care, particularly in HICs and middle-income countries, suggest the influence of SES on stroke outcomes may have shifted. As a result, the relationships observed in previous reviews may no longer hold, highlighting the need for an updated analysis. We have thus adapted methodologies to incorporate recent findings, summarize new trends in stroke-related inequalities, and examine the factors driving them.

SES describes a person’s social standing or class within society; yet this definition is difficult to measure.^3^ Consequently, many individual proxies, such as education, income, and occupation, as well as composite or group proxies, such as neighborhood SES, housing quality, and crime rates, have been used to approximate SES in research (Table).^4^ This variety of proxies, combined with variations in their definition across different countries, often prevents direct data comparisons between cohorts.

**Table.**
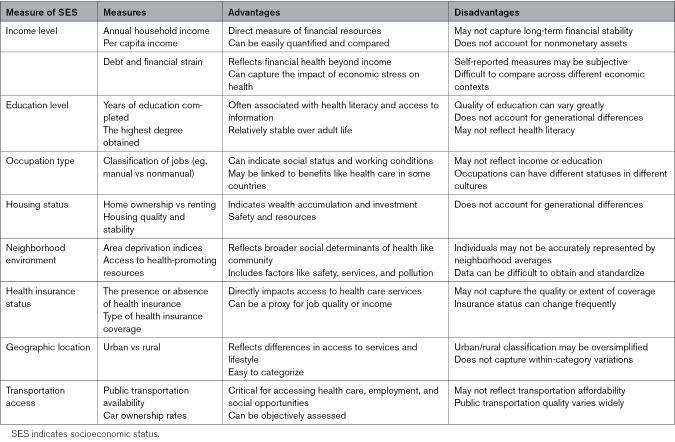
Measurements of SES in the Literature About Stroke

This study aims to provide a narrative review of the influence of SES on stroke, following a timeline structure of stroke: it begins by addressing stroke occurrence, access to and quality of acute care, recurrence, disability, and mortality. For recurrence and mortality and disability, we define long-term outcomes as those occurring after 1 year following the stroke.^5^ Within these sections, the interaction of SES with sex and ethnicity is also considered. By interaction, we refer to the combined effects that collectively shape outcomes differently than when each factor is considered independently. As shown in Figure 1, this approach aims to outline the drivers of inequalities comprehensively.

**Figure 1.**
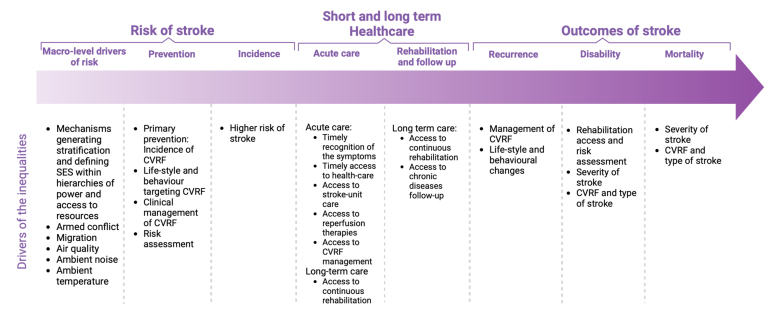
**Timeline of stroke risk and outcomes: key determinants influencing health inequalities.** Stroke risk and outcomes result from a complex, progressive process where an individual’s biopsychosocial characteristics interact with their environment. Factors at various levels of this relationship influence each other in a reciprocal, longitudinal system, where the preceding ones shape each stage. CVRF indicates cardiovascular risk factor; and SES, socioeconomic status.

## Methods

**Figure 2.**
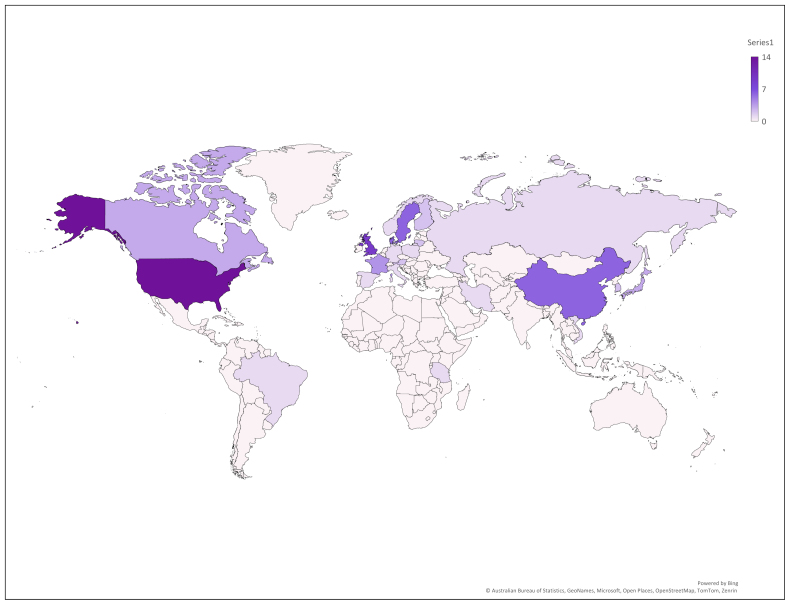
**Global distribution of evidence on socioeconomic status (SES) and stroke.** Heatmap shows the global distribution of reviewed evidence on the relationship between SES, stroke risk, and outcomes. Darker shades indicate countries with more cohort studies, while lighter shades reflect fewer studies.

We searched PubMed for publications from January 2015 to July 2024 including the terms “stroke,” “cerebrovascular accident,” “cerebrovascular disorders,” “cerebral haemorrhage,” “subarachnoid haemorrhage,” “socioeconomic status,” “social determinants,” “social determinants of health,” “education,” “income,” “gender,” “occupation,” “poverty,” “inequality,” and “deprivation,” without restrictions on language. We included prospective and retrospective, nationwide, population-based, international, or multicohort analyses of routine data sources demonstrating a link between SES and stroke incidence, severity, care, and outcomes. This search strategy resulted in 68 studies, mainly from HICs and middle-income countries, as portrayed in Figure 2 and detailed in the Table S1. This synthesis provides an updated understanding of SES-related disparities in stroke, informing health care strategies and interventions in the context of governmental policies on inequalities such as education, poverty, and climate emergency.

### SES and Stroke Risk

The 2019 GBD study (Global Burden of Disease) highlighted a 70% increase in global incident strokes from 1990 to 2019.^6^ While age-standardized incidence rates decreased, age-specific incidence among those under 70 years of age rose by 15%.^6^ Globally, the stroke burden declined, driven mainly by HICs, while upward age-standardized incidence rate trends were concentrated in regions with moderate or low Social Development Index.^6^ In LMICs, rising incidence is compounded by an aging population and a shift from infectious to chronic diseases over the past 30 years.^7^

**Figure 3.**
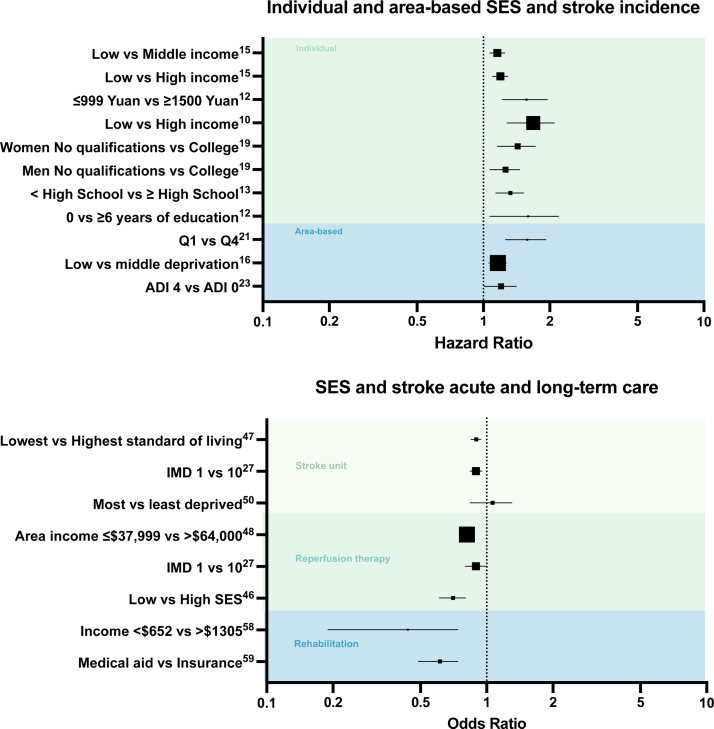
**Forest plots illustrating the association between socioeconomic status (SES) and stroke incidence and care.** Forest plots illustrate the association between SES and stroke incidence and acute and long-term stroke care outcomes. The upper part compares individual SES factors (eg, income, education) and area-based SES factors (eg, neighborhood deprivation, Area Deprivation Index [ADI]) on stroke incidence, represented as hazard ratios (HRs). The lower part shows the relationship between SES and access to stroke care, with odds ratios (ORs) reflecting disparities in acute and long-term care (eg, admission to stroke units and rehabilitation therapy). Squares represent point estimates, and error bars indicate 95% CIs. The size of each square is proportional to the sample size or weight of the study in the analysis. IMD indicates Index of Multiple Deprivation.

The 2015 review found an inverse association between SES and stroke incidence, particularly for ischemic stroke.^2^ Nine nationwide, 11 population-based, and 2 international studies confirm this finding.^8–29^ These studies, conducted across the United States, United Kingdom, Europe, Japan, Australia, Finland, China, and Korea, used various SES proxies, including education, income, health insurance, and area-based indicators like neighborhood SES and the Index of Multiple Deprivation.^8,10–28^ Results are shown as ratios and 95% CIs in Figure 3.

Substantial evidence links low education with a higher risk of stroke in the United States (low versus high education: hazard ratio [HR], 1.32 [1.14–1.52]), Australia (no qualifications versus college: HR, 1.21 [0.97–1.51]), Europe (less versus most educated men: relative index of inequality, 1.54 [1.25–1.91]), and rural China (0 versus ≥6 years of education: HR, 1.53 [1.07–2.19]).^12,13,18,19,25^ This association is more pronounced for ischemic stroke than for other subtypes (0 versus ≥6 years of education; ischemic HR, 1.63 [1.07–2.47]; nonischemic HR, 1.29 [0.64–2.63]).^12^ The ARIC study (Atherosclerosis Risk in Communities) in the United States (n=11 509) found the highest ischemic stroke risk in those with basic education (low versus advanced education: adjusted HR [aHR], 1.31 [1.09–1.61]) but not for hemorrhagic stroke (aHR, 1.02 [0.55–1.85]).^11^ Lower income is also associated with higher stroke risk in the United States (low versus higher income: HR, 1.59 [1.41–1.79]), China (aHR, 1.67 [1.28–2.12]; ≤999 versus ≥1500 Yuan; aHR, 1.54 [1.22–1.95]), and Finland (aHR, 1.72 [1.62–1.79]).^10,12,13,18^ This association exists for both ischemic and hemorrhagic strokes in China and Finland.^10,12^ A nationwide Korean study found similar associations (middle versus high income: HR, 1.15 [1.07–1.25]; low versus high income: HR, 1.19 [1.10–1.29]).^15^ Additionally, an ARIC cohort study (n=8989) reported that an income drop increased stroke risk (HR, 1.69 [1.35–2.11]), though the association weakened after adjusting for CVRF.^20^ Other SES proxies linked to stroke incidence included health insurance and occupation. In the United States, lack of insurance was associated with a higher stroke risk (HR, 1.46 [1.16–1.84]), and in Japan, a nationwide study found that professional workers had a higher stroke risk than blue-collar workers, even after adjusting for smoking and alcohol consumption (aHR, 3.79 [1.17–1.42]).^10,14^

Area-based SES proxies, like neighborhood deprivation, were linked to increased stroke risk in nationwide studies from Sweden and United Kingdom and population-based studies from the United States and Japan.^8,16,17,21,23,27^ Both Japanese and UK Sentinel Stroke National Audit Programme data reported higher stroke risk in deprived versus less-deprived areas (Japan: aHR, 1.19 [1.01–1.41]; UK: adjusted incidence rate ratios, 2.0 [1.7–2.3] for ischemic stroke and 1.6 [1.3–1.9] for intracerebral hemorrhage).^23,27^ In United Kingdom, this relationship was intensified by comorbidities, with adjusted incidence rate ratios of 3.2 (2.7–3.8) for diabetes, 2.3 (1.9–2.8) for hypertension, and 1.5 (1.2–1.9) for atrial fibrillation.^27^ In a nationwide study in Sweden, high neighborhood SES was protective against stroke (HR, 0.87 [0.78–0.96]), while low SES increased risk (HR, 1.16 [1.06–1.27]).^16^ A sibling analysis in the same cohort confirmed this relationship, even after controlling for individual SES and comparing genetically related siblings in different SES neighborhoods, suggesting nongenetic mechanisms.^16^ The US REGARDS study (United States Reasons for Geographic and Racial Differences in Stroke) also found higher risk in deprived neighborhoods (defined using a composite measure that includes household income, the proportion of adults, educational attainment, and occupation), unmitigated by adjusting for CVRF (HR, 1.56 [1.26–1.92]).^21^

Concerning the interaction between SES and ethnicity, the US REGARDS cohort did not find differential rates of stroke between Black and White individuals.^21^ In contrast, NOMAS (Northern Manhattan Study; n=3298) found an increase in incidence among Black, Hispanic, and White individuals (incidence per 1000 person-years: 13 [Black individuals], 10 [Hispanic individuals], and 9 [White individuals]).^22^ In both cohorts, SES and CVRF modified the risk.^21,22^ In the NOMAS cohort, adjusting by these factors lessened the disparity between Black and White individuals (HR, 1.51 versus aHR, 1.33) and fully explained the disparity between Hispanic and White individuals (HR, 1.48; aHR, 1.07).^22^

Incidence in both sexes in United Kingdom, France, Australia, and China was influenced by SES, with women and men from lower SES having a higher risk of stroke.^8,24,29^ In United Kingdom, an analysis of the UK Biobank data (n=471 971) found that women from low SES had a higher risk of stroke than men from low SES (aHR, 1.22 [1.06–1.41]).^24^ Further, this disparity in incidence between women and men was diminished by adjusting by CVRF.^8^ In Australia, a population-based analysis (n=253 657) found lower education was associated with higher stroke rates for both sexes, yet only significant for women when accounting for CVRF (HR, 1.26 [1.04–1.54]), similar to a Chinese study (n=2852) in which low education was associated with higher risk only for women (low versus high education in women: HR, 3.68 [1.70–7.97]; low versus high education in men: HR, 0.93 [0.44–1.98]).^19,28^

In conclusion, research over the past decade reaffirms the persistent association between lower SES and increased stroke risk, particularly for ischemic strokes.^10–15,18–20,25,26^ CVRF appears to mediate this association.^16,18,19,23,27^ Additionally, disparities in stroke incidence according to ethnicity and SES remain significant.^21,22^ In the NOMAS study, SES did not fully explain the differences in stroke rates between Black and White individuals; however, disparities between Hispanic and White individuals were entirely accounted for by SES and CVRF.^21,22^ The evidence supporting the association between education and income with ischemic stroke is robust. While the relationship between education and hemorrhagic stroke remains unclear, lower income is associated with a higher risk of hemorrhagic stroke.^10,12^

### Population-Level Determinants of Stroke Risk

Population-level determinants of health can be understood as structural drivers of health.^30^ These generate stratification and divide the society into social classes, defining individual SES and hierarchies of resource access.^30^ These conditions, such as armed conflict, migration, and environmental factors, tend to disproportionately affect those with lower SES, while also contributing to further socioeconomic disadvantage. Thus, the relationship between these determinants and individual SES is typically bidirectional.^30^ During this decade, available research has primarily focused on armed conflict, migration, and environmental conditions.

#### Armed Conflict and Migration

Armed conflict increases cardiovascular disease rates directly through violence and stress and indirectly through migration.^31^ The Vietnam Health and Aging Study (n=2447) found no statistically significant association between wartime stress and stroke risk (adjusted odds ratio [aOR], 1.04 [0.96–1.15]), furtherly mitigated by higher education (aOR, 0.58 [0.29–1.17]) and amplified in those with a professional degree compared with agricultural workers (aOR, 1.95 [0.88–4.30]), suggesting SES modulates the violence-stroke risk relationship.^32^ About migration, 5 population studies from Denmark and Canada explored the interaction between migration and SES.^33–37^ Ontario data (n=8 million) showed lower stroke rates in immigrants, primarily from Western countries, compared with long-term residents (ischemic stroke: HR, 0.71 [0.69–0.72]), even after adjusting for SES.^36^ However, this study likely excluded the most vulnerable migrants, excluding individuals not enrolled in a health insurance plan, as it relied on a registered people database.^36^ Analysis from the same cohort found that long-term Ontario residents had higher 5-year mortality than immigrants (46.1% versus 64.5%, respectively), but this disparity reversed when accounting for CVRF and SES (HR, 0.94 [0.88–1.00]), highlighting the impact of these factors on the Healthy Migrant Effect.^37^

In Denmark, population-based analyses (n=132 936) found higher stroke risk in both Western (German, Norwegian, Swedish, and Polish: IRR, 2.25) and non-Western immigrants (Turkish, Iraqi, Pakistani, ex-Yugoslavian, and Iranian: IRR, 1.37) compared with Danish-born individuals.^33^ One-year all-cause mortality after ischemic stroke was similar for migrants and Danes but higher for Western immigrants after hemorrhagic stroke (odds ratio [OR], 1.46 [1.02–2.10]), an association that disappeared after adjusting for education and occupation.^33^ Non-Western women initially showed lower hemorrhagic stroke mortality, but this difference also disappeared after adjusting for occupation and income.^33^ Despite similar education and income, immigrants were less likely to be admitted to stroke units (81.5% versus 83.9%) and had lower odds of receiving reperfusion therapy (aOR, 0.67 [0.49–0.92]).^34,35^ While this did not affect mortality, its impact on disability remains unclear.

#### Environmental Conditions

**Figure 4.**
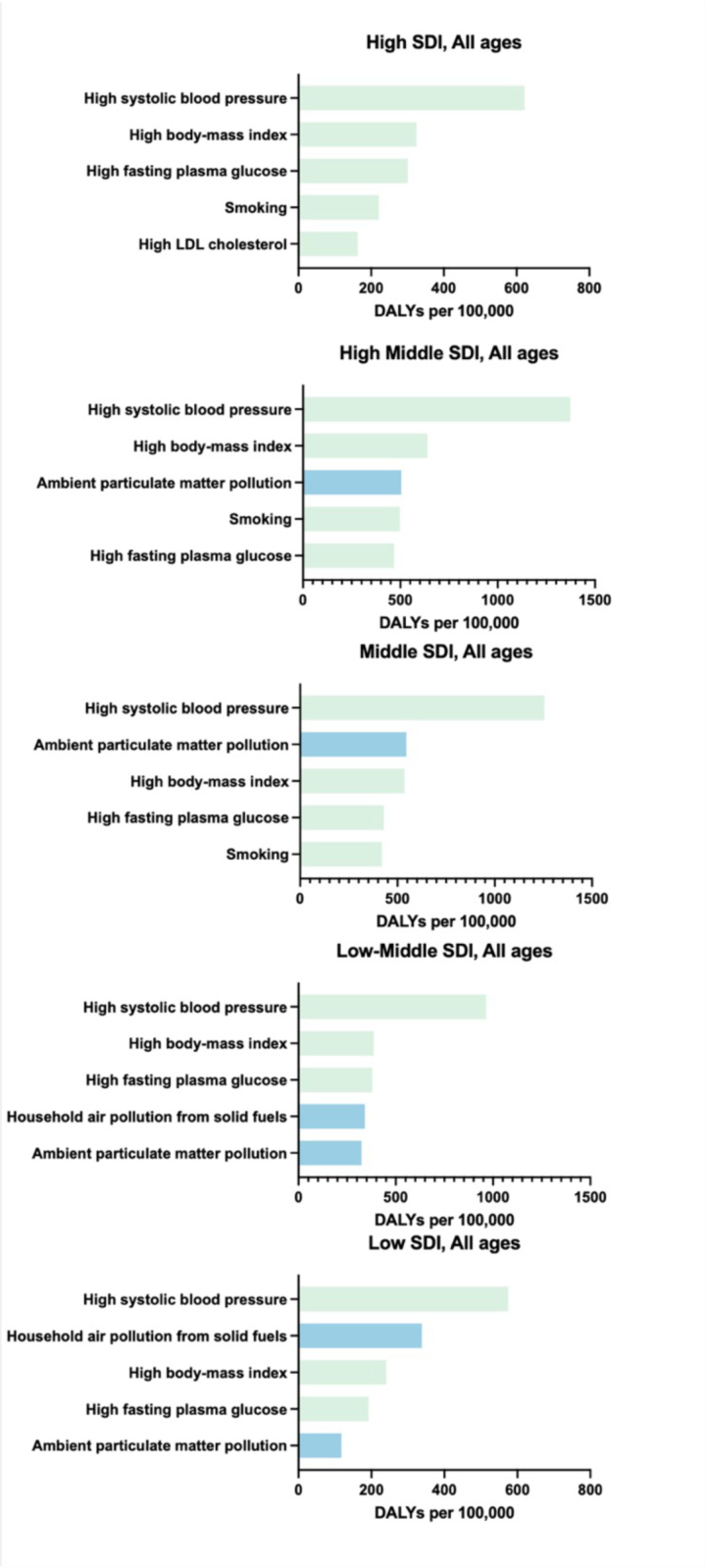
**Risk factors for stroke according to Social Development Index (SDI).** This figure, based on the GBD study (Global Burden of Disease) data reported by Feigin et al, shows the top stroke-related risk factors (disability-adjusted life-years [DALYs] per 100 000) across different SDI levels. High SDI: stroke burden is mainly driven by high systolic blood pressure, high body mass index, fasting plasma glucose, smoking, and high LDL (low-density lipoprotein) cholesterol. High-middle and middle SDI: in addition to cardiovascular risks like high systolic blood pressure and body mass index, ambient particulate matter pollution becomes a significant factor. Low-middle and low SDI: environmental risks, particularly household air pollution and particulate matter, combine with cardiovascular risks to increase stroke burden. Bar lengths indicate the DALYs contributed by each risk factor in each SDI group.

The 2019 GBD study highlighted stroke risk factor differences across Social Development Index tiers, with high Social Development Index areas linked to lifestyle and CVRF and middle-to-low Social Development Index regions facing additional risks from environmental hazards like pollution and solid fuel exposure (Figure 4).^38^ While evidence on environmental impacts on stroke risk is widely available, the SES interaction needs further clarification.^39^ We reviewed 6 studies on SES-environment interactions (2 on air pollution, 2 on noise, and 2 on temperature, including a meta-analysis).^40–45^

Exposure to ultrafine particles, elemental carbon, and NO_2_ increases stroke risk.^41^ Danish nationwide and US population-based data found increased incidence in deprived versus less-deprived populations when exposed to the same pollutants (Denmark: low versus high education, 568 versus 423 strokes per 100 000 person-years; United States: more versus less disadvantaged: IR, 1.24 [1–1.55]), showing a synergism between air pollution and SES in increasing stroke risk.^40,41^

Long-term noise exposure also raises stroke risk.^42^ Over 2 decades of data from 9 Scandinavian cohorts showed that each 10-dB increase in long-term exposure to transport noise resulted in a 6% increase in stroke risk, not modified by education level.^42^ A nationwide analysis from Switzerland found higher cardiovascular mortality in low-income people exposed to transportation noise.^43^ Yet, no differences in specific stroke-related mortality were found.^43^

Last, a large global meta-analysis on ambient temperature, defined as the external or outdoor temperature, measured in degrees Celsius, showed that high and low ambient temperatures were associated with stroke morbidity and mortality.^45^ When analyzing by socioeconomic strata, the effect of higher heat was the greatest in HIC versus LMIC (relative risks [RRs], 1.16 [1.00–1.34] and 1.14 [0.97–1.33]).^45^ Cold-related stroke morbidity and mortality risks were the highest in LMICs compared with HICs.^45^ Similarly, a Seoul emergency department–based study (n=63 564 emergency visits) noted a slight rise in summer stroke visits among medical aid beneficiaries versus the insured (RR, 1.05 [1–1.106]).^44^

Overall, recent literature suggests that the interaction between SES and environment compounds stroke risk, especially in disadvantaged populations. As the climate emergency intensifies and SES disparities widen, this topic will likely become a major focus of future research.

### Stroke Health Care and Clinical Management

The 2015 review linked lower SES with inadequate health care access.^2^ This update includes nationwide studies from United Kingdom, Sweden, France and the United States, along with population-based and multicenter data from Canada and Japan that examine the persistence of this association.^27,46–54^ Results are shown as ratios and 95% CIs in Figure 3.

#### Acute Care

Since the 1990s, stroke units have been crucial in reducing mortality and dependence.^55^ However, access to acute care is unequal around the world.^56^ In LMICs, delays in recognition and treatment of strokes have been recognized.^56^ In Ethiopia, urban patients arrived to the hospital after a median time of 17 hours, whereas rural patients arrived to the hospital after a median of 60 hours.^57^ In rural areas, medical services are often not equipped to diagnose and treat acute stroke patients, and they are more likely to be treated by internists or family physicians.^56^

In Ontario (n=11 050), wealthier patients had better access to stroke units and neurology consultations, despite publicly funded health care (stroke units, 66.7% versus 63.8%; neurology consults, 75% versus 68.1%; *P*=0.02 and *P*<0.001).^49^ Nationwide French data, with universal health care, aligned with this finding, showing similar disparities (the lowest versus the highest quartile of living standard: RR, 0.90).^47^ The Sentinel Stroke National Audit Programme data in United Kingdom also found that deprived patients were less promptly admitted to stroke units (the most- versus the least-deprived adjusted incidence rate ratio, 0.89 [0.84–0.95]).^27^

Low-income populations in Ontario arrived within 2 hours of symptoms less often than high-income ones (31.2% versus 37.8%; *P*<0.001).^49^ Disparities in reperfusion therapy access between income groups (12.8% versus 14.6%) disappeared when adjusting to clinically eligible status (35.6% versus 33.9%).^49^ In Japan, the Kochi Acute Stroke Survey (n=9651) found that people from deprived areas delayed care seeking by >2 hours (OR, 2.04 [1.30–3.26]), but this did not affect reperfusion therapy access.^50^ In contrast, nationwide data from the United States, United Kingdom, and Sweden showed reduced reperfusion therapy access for lower-income groups (low versus high income: OR, 0.81 [0.78–0.85] in the United States; aOR, 0.89 [0.80–0.99] in United Kingdom; OR, 0.7 [0.61–0.80] in Sweden), even after adjusting for stroke severity.^27,46,48^ However, a nationwide Swedish mediation analysis found that these disparities did not significantly impact survival, with CVRF and stroke severity primarily driving mortality.^46^

SES and ethnicity overlap in contributing to disparities in reperfusion therapy, as shown in nationwide cohorts.^51,52^ A 2016 nationwide US analysis found that Black and Hispanic patients were less likely than White patients to receive thrombolysis (aOR, 0.84 [0.76–0.92] and 0.85 [0.75–0.96], respectively).^51^ Two other US national cohorts confirmed these disparities (Black versus non-Hispanic White individuals: tissue-type plasminogen activator: OR, 0.85 [0.80–0.91]; mechanical thrombectomy: OR, 0.75 [0.70–0.82]).^52,54^ In United Kingdom, the South London Stroke Register found that, after adjusting for sex, SES, and stroke severity, Black patients had higher odds of being admitted to stroke units (OR, 1.76 [1.35–2.29]).^53^

#### Long-Term Care

A population-based cohort from Taiwan found that low SES was associated with reduced inpatient rehabilitation 7 to 12 months after stroke (low versus medium SES: OR, 0.38 [0.19–0.74]).^58^ In Korea, a population-based retrospective cohort from the National Health Insurance Service reported similar disparities.^59^ Rural areas (rural versus urban: OR, 0.745 [0.66–0.84]), medical aid insurance (OR, 0.61 [0.49–0.74]), and low income in mild-to-moderate strokes (OR, 1.51 [1.11–2.1]) were associated to lower rehabilitation therapy rates.^59^

In conclusion, care disparities persist even within the publicly funded health care systems in countries such as Canada, United Kingdom, France, and Sweden. While SES is associated with differences in care-seeking behavior and the likelihood of arriving within 2 hours of symptom onset, its effect on access to reperfusion therapy remains unclear. Access to stroke units continues to be unequal, and the full impact of these disparities on outcomes requires further investigation.

### SES and Stroke Outcomes

#### Stroke Recurrence

**Figure 5.**
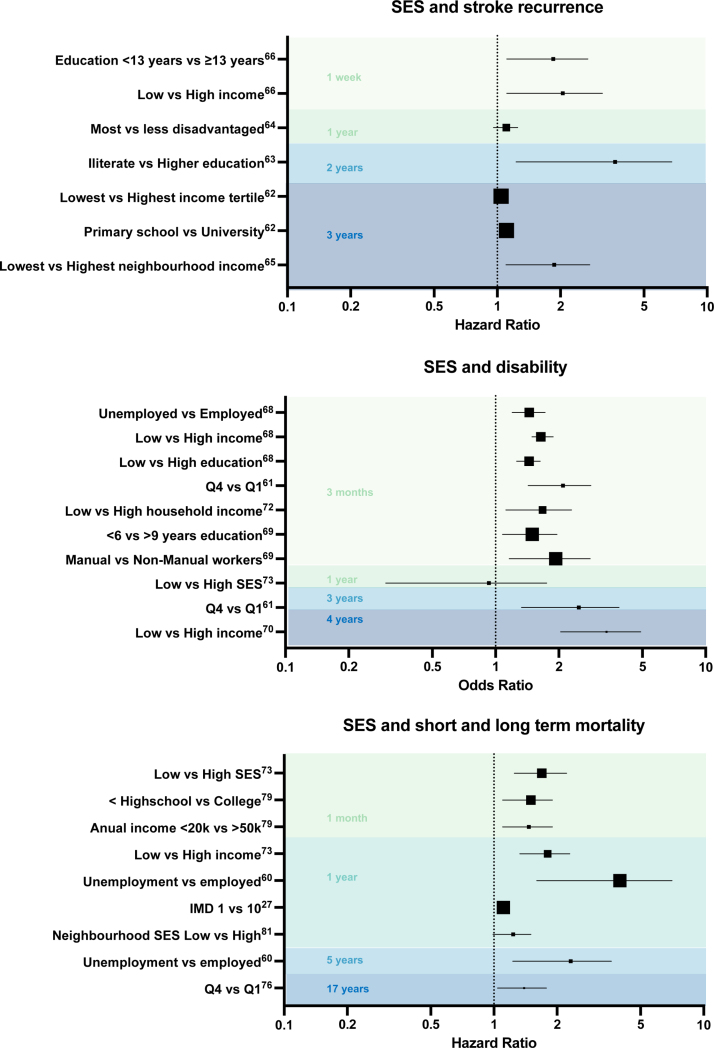
**Forest plots illustrating the association between socioeconomic status (SES) and stroke incidence and care.** Forest plots illustrate the impact of SES on stroke recurrence, disability, and mortality. In the top part, the hazard ratios depict stroke recurrence across various SES measures over different follow-up periods, ranging from 1 week to 3 years. The middle part shows odds ratios for disability based on SES factors, with follow-up periods spanning from 3 months to 4 years. The bottom part displays hazard ratios for short- and long-term mortality, reflecting the influence of SES at follow-up intervals ranging from 1 month to 17 years. The error bars represent 95% CIs, with larger squares indicating studies that included greater sample sizes. IMD indicates Index of Multiple Deprivation.

The 2015 review highlighted the need for more research on the SES-stroke recurrence association.^2^ This update includes 7 large-scale studies—an increase from the previous decade but still limited compared with research on other outcomes.^60–66^ Results are shown as ratios and 95% CIs in Figure 5.

The revised literature shows no consensus on defining stroke recurrence. Most studies defined it as focal neurological symptoms occurring 24 hours after the initial event, excluding edema, mass effect, brain shift, or hemorrhagic transformation.^60^ However, some methodologies could not identify a second stroke within 28 days due to delayed follow-up, and others only counted recurrences post-hospital discharge.^62,64,65^ Stroke recurrence should also be interpreted in context, as higher survival rates and life expectancy expose individuals to more subsequent strokes, meaning higher recurrence rates may reflect improved survival.

Recurrence has been associated with income and education, both individually and as a composite measure.^60–64^ A Swedish nationwide study, which defined recurrency as a new event after 28 days of the first stroke and had a 3-year follow-up, found that lower education and income were associated with higher recurrence risk after adjusting for CVRF (primary versus university: HR, 1.10 [1.06–1.16]; the lowest versus the highest income tertile: HR, 1.05 [1.0–1.08]), though this risk decreased over time.^62^ Similarly, a US population-based study (n=2125), which defined recurrence as a new stroke occurring after discharge from the initial stroke hospitalization, reported that low neighborhood SES (HR, 1.74 [1.10–2.76]) and Black ethnicity (HR, 1.31 [1.01–1.70]) were associated with higher recurrence risk, but these associations lost significance after adjusting for CVRF.^65^

In China, lower education was consistently linked to higher ischemic stroke recurrence, even after adjusting for CVRF in a 2-year follow-up (illiterate: HR, 2.90 [1.24–6.79]; primary: HR, 2.59 [1.17–5.77]; middle: HR, 2.34 [1.08–5.08] versus higher education).^63^ In this analysis, recurrence was defined as a deterioration of the previous neurological exam or new symptoms in a new vascular territory.^63^ The Nanjing-Registry similarly found that lower income (HR, 1.87 [1.11–3.17]) and education (HR, 1.73 [1.11–2.70]; education, <13 versus >13 years) were associated with higher recurrence rates, defined as new events after 7 days of the index event, after adjusting for CVRF.^66^

In contrast, studies from Iran (n=684) and Australia (n=25 421) showed no clear SES-stroke recurrence correlation (HR, 1.10 [0.96–1.25]).^60,64^ This lack of association may be due to higher mortality rates among the most disadvantaged individuals after their first stroke, limiting the likelihood of experiencing a recurrence.^60,64^

In summary, despite increased research, ambiguities regarding SES’s influence on stroke recurrence and its underlying mechanisms persist. Although evidence remains limited, 3 included studies adjusted for CVRF, partly explaining the association. However, further exploration of the mechanisms and the need to control for mortality in these cohorts remain important areas for future research.

#### Disability

The 2015 review linked low SES to severe strokes and poor 1-year outcomes, but the longer-term effects were unclear.^2^ In this update, we analyzed studies with varying follow-up periods, including both short-term (3 months) and long-term follow-ups ranging from 3 to 5 years, including 3 nationwide, 3 population-based, and 3 multicenter studies.^60,61,63,67–72^ Most studies used the modified Rankin Scale to evaluate functional outcomes, except for 2 that used activities of daily living.^67,71^

At 3 months, the China National Stroke Registry found that manual occupations and lower education levels were associated with a higher modified Rankin Scale score (manual versus nonmanual workers: aOR, 1.81 [1.16–2.82]; <6 versus >9 years of education: aOR, 1.45 [1.08–1.96]).^69^ Another Chinese multicenter study (n=3975) linked low household income to increased disability at 3 months (low versus high income: OR, 1.60 [1.12–2.31]).^72^ In United Kingdom, the South London Stroke Register identified greater disability for ischemic stroke among the most deprived (OR, 2.01 [1.43–2.84]), particularly among women and older individuals, but not for hemorrhagic stroke.^61^ Similarly, a Danish nationwide study found that lower education, income, and unemployment were associated with higher disability (OR, 1.44 [1.26–1.63], 1.59 [1.49–1.88], and 1.42 [1.2–1.72], respectively), explained by CVRF and lifestyle factors.^68^ Finally, the US BASIC project (United States Brain Attack Surveillance in Corpus Christi) linked deprived neighborhoods (defined by a composite measurement of household income, education, and occupation of the people living in it) to higher disability at 3 months, even after adjusting for individual SES (mean difference, −0.27).^71^

At 1 year, the ZanStroke cohort (n=720) in Zanzibar found no association between SES and functional outcomes at 1 year poststroke.^73^ However, this cohort had a 38% first-year mortality rate, with severe strokes likely represented in fatalities, indicating survival bias.^73^ In Iran, low SES was linked to greater disability at 1 year (probability of modified Rankin Scale score, <2; the highest versus the lowest SES: aOR, 0.29 [−0.57 to 1.16]).^60^

The relation between SES and disability seems mediated by CVRF. Two nationwide Danish studies found that stroke severity, which itself was influenced by CVRF, explained this association.^67,68^ The Iranian cohort analysis also found that adjusting for stroke severity and CVRF eliminated the SES disability link at 1 and 5 years.^60^

The last decade of research has extended follow-up periods. For instance, the South London Stroke Register’s 3-year follow-up aligned with earlier findings, showing higher disability risk in the most deprived quartile (aOR, 2.27 [1.33–3.87]).^61^ As before, this association was found for ischemic but not for hemorrhagic stroke.^61^ In China, a population-based cohort study found income was a strong predictor of long-term disability at 4 years, even after adjusting for employment and CVRF (low versus high income: OR, 3.17 [2.04–4.91]).^70^ The Mashhad Stroke Incidence Cohort in Iran was the most extended follow-up and showed higher disability risk for lower SES during the first year, which did not persist at 5 years.^60^

In summary, the last decade of research continued to find an association between lower SES and increased disability. While our previous review established this association up to 1 year after stroke, this update found evidence that this relationship persists up to 4 years after stroke. The association appears to be stronger for ischemic stroke than for hemorrhagic stroke and seems to be mediated by CVRF and stroke severity.

#### Mortality

The 2015 review found that lower SES was linked to increased short-term mortality (in-hospital and up to 1 year poststroke), while the long-term impacts (beyond 1 year) remained unclear.^2^ This update includes studies with extended follow-up periods, including cohorts with 3, 10, and 17 years of data on mortality.^60,74–76^

The association between SES and mortality has been reinforced in understudied populations, like Brazil, and supported by studies from United Kingdom and the United States.^77^In Brazil, women with <8 years of education had higher stroke mortality, regardless of state development (RRs, 4.59 [3.26–6.47] in least developed states and 3.31 [3.04–3.59] in highly developed states).^77^In United Kingdom, women initially had higher poststroke mortality (IRR, 1.36 [1.32–1.40]) compared with men, but this reversed after adjusting for Index of Multiple Deprivation (RR, 0.96 [0.93–0.98]), suggesting greater vulnerability to socioeconomic disadvantages.^8^ In the United States, Black patients had higher in-hospital mortality compared with White patients, even after adjusting for SES and comorbidities (aOR, 0.71 [95% CI, 0.64–0.78]), while SES explained the mortality difference between Hispanic and White patients.^51^

First-month mortality after stroke was linked to lower income in Catalonia, Zanzibar, and the United States. In Catalonia (n=16 344), lower-income individuals had higher mortality after ischemic stroke (OR, 1.60 [1.19–1.55]), though this association weakened after adjusting for CVRF.^78^ The ZanStroke cohort also showed lower survival for the most deprived (highest to lowest: HR, 0.64 [0.44–0.94]).^73^ Similarly, the US REGARDS cohort found higher 1-month mortality rates associated with lower education and income (less than high school versus college graduate: RR, 1.5 [1.1–1.9]; income <20k versus >50k: RR, 1.4 [1.1–1.9]).^79^ In a nationwide study in Sweden, low-income patients had a higher mortality risk after hemorrhagic (3.2% increase) and ischemic stroke (1% increase), with roughly half of the effect driven by increased stroke severity.^46^

One-year mortality has been linked to low income and education. The ZanStroke cohort found higher 1-year mortality in the lowest income group (the highest versus the lowest SES: HR, 0.56 [0.42–0.75]), while the Mashhad cohort in Iran identified unemployment as a risk factor for increased mortality at 1 and 5 years (high to low SES: aHR, 0.84 [0.65–1.09] and 0.56 [0.42–0.75]).^60,73^ The China Stroke Register also found that low education was associated with higher mortality, even after adjusting for CVRF (OR, 1.25 [1.05–1.48]).^80^ Similarly, data from United Kingdom and the United States showed higher mortality risk in deprived areas (aHR, 1.22 for the United States and 1.11 for United Kingdom), even after adjusting for comorbidities.^27,81^ In United Kingdom, the SES indicator was the Index of Multiple Deprivation, while in the United States, a composite measure including social ties, neighborhood income, poverty, and demographic composition was used.^27,81^ Social ties, poverty reduction, and residential stability were protective against mortality.^81^

At 3 years, data from China and Catalonia showed higher mortality risk in lower-income individuals (HR, 5.35 [2.95–9.70] in China; aHR, 1.68 [1.44–1.96] in Catalonia).^74,78^ After 5 years, the link between lower education and higher mortality persisted in Iran (aHR, 1.84 [95% CI, 1.05–3.23]).^60^ Longer follow-up periods in recent studies have provided valuable insights, such as a 10-year study in Korea showing better long-term survival with premium health insurance compared with public aid.^75^ A 17-year follow-up in the South London Stroke Register revealed increased mortality with greater socioeconomic deprivation, particularly for intracranial hemorrhage (HR, 1.36 [95% CI, 1.04–1.78] for the most-deprived-area quartile).^76^ Data from United Kingdom and Catalonia showed that the relationship between SES and mortality is modified when accounting for CVRF, with the UK Sentinel Stroke National Audit Programme data indicating a change in SES and 1-year mortality and Catalan data showing a decreased association after 36 months.^27,78^

In summary, the association between SES and short-term mortality has been reaffirmed and now described in understudied populations in middle-income countries. Studies suggest CVRF and stroke severity influence this relationship. The value of recent research lies in longer-term follow-ups, which consistently support this association, especially between income and mortality, for both ischemic and hemorrhagic stroke. Further studies will provide more definitive evidence on long-term outcomes and underlying mechanisms.

## Conclusions and Future Directions

This review updates on the global association between SES and stroke (prevention, clinical care, and outcomes) since 2015, highlighting persistent disparities.^6,7^ It includes extended follow-up periods of up to 17 years, offering insights into long-term stroke outcomes, particularly in understudied populations like those in LMICs. Additionally, it explores the role of environmental factors such as air pollution, noise, and temperature, which were not covered in previous reviews. As HICs and LMICs face rising CVRFs linked to changing lifestyles and aging populations, additional global risks like air quality and climate change impose further burdens. Although environmental risk affects all countries, LMICs bear a disproportionate burden of their impact.^31,32^ In addition, in LMICs, the coexistence of violence, trauma, and transmittable diseases with the increase of chronic and nontransmittable diseases overburdens health care systems. While establishing evidence-based care, including stroke unit care, is cost-effective in the medium and long term, it is also costly and resource intensive to implement.^27,29,47,82^

Recent research highlights the impact of SES on stroke risk and outcomes, improving upon the 2015 data with extended follow-up periods.^60,75,76,80^ Although evidence on stroke recurrence has increased, it remains insufficient; detailed long-term studies with standardized definitions of recurrence are crucial to fully understand SES-related trends.^60–63^

The recent literature marks a shift from merely describing disparities to actively exploring the underlying mechanisms.^83^ Recent research methodologies such as mediation analysis have reaffirmed 2 key factors: (1) CVRF and (2) access to health care.^46,67^ CVRF that accounts for severe strokes, such as large vessel atherosclerosis and atrial fibrillation, might be key in mitigating incidence, severity, and disability.^67^ Access to health care, particularly stroke units, has also emerged as a driver of disparities; yet comprehensive data on stroke unit availability and equality of access are limited.^27,47,49,82^ The number of studies on reperfusion therapies has grown, showing unequal access influenced by SES and race/ethnicity and often associated with delayed consultation.^27,48,50,67^ While mediation analysis suggests that improving access to stroke units and reperfusion therapies might result in smaller decreases in absolute risk differences, the prevention and modification of CVRFs take time to show significant impacts on reducing risk, disability, and mortality from stroke.^67^ As the cardiovascular landscape evolves, it remains essential to make basic stroke unit care, reperfusion therapy, neuroimaging, and rehabilitation more accessible.^67^

There is a need to define SES and its measures and to conduct population-based or nationwide research in low-income countries and LMICs, including detailed long-term follow-up for recurrence and disability and investigation of the postconflict/migration impact on stroke outcomes. This is a resource-intensive task and needs political and clinical cooperation to be achieved. One proposed approach is an international framework for measuring SES, aiming to facilitate global comparisons.^84^ However, SES means different things in different countries and is influenced by cultural norms, geographic contexts, and changes over time. While harmonization efforts are valuable, flexibility and context-specific understanding are essential to capture the full range of socioeconomic influences on health. Researchers should aim to clearly define and justify the SES proxies used in their studies to enable more transparent comparisons across the literature.

Additionally, SES-adjusted studies on the climate emergency are needed. Research gaps also exist for the LGBTQ+ community, hormone therapy recipients, and individuals living with multimorbidity and specific comorbidities that are known to increase cardiovascular risk, such as HIV/AIDS, in which SES might be an influential factor, especially relevant for LMICs. Future research should continue focusing on the underlying mechanism driving SES association with stroke, particularly by integrating research methodologies capable of explaining mediation and direct effects, given SES’s multidimensionality and stroke’s cumulative nature of lifelong exposures. On a population level, it is crucial to prioritize the gathering of quality information on LMICs, as most current research derives from HICs and does not adequately reflect the LMIC populations that bear a greater burden of stroke.^6^ Additionally, integrating data on environmental factors such as air pollution, noise, and temperature with SES will be essential for formulating public policies tailored to mitigate risk factors in vulnerable groups. Clinical efforts should concentrate on managing vascular risk factors with a particular focus on socioeconomically more deprived groups to reduce disparities in stroke incidence and severity, which will directly influence survival rates and reduce disability. The inequalities in stroke incidence and outcomes are still present, and despite significant efforts, new challenges have risen that demand sustained and coordinated efforts to address them effectively.

## Article Information

### Acknowledgments

Dr Pantoja-Ruiz, Dr Akinyemi, Dr Lucumi-Cuesta, D. Youkee, E. Emmett, Dr Kalansooriya, Dr Soley-Bori, Dr Wolfe, and Dr Marshall designed and produced the review outline. Dr Pantoja-Ruiz searched the scientific literature and with Dr Marshall assessed articles for inclusion. Drs Pantoja-Ruiz, Lucumi-Cuesta, and Marshall produced the initial draft of the manuscript and figures, which Dr Akinyemi, E. Emmett, D. Youkee, Dr Lucumi-Cuesta, Dr Kalansooriya, Dr Soley-Bori, and Dr Wolfe refined further. All authors critically revised several drafts and approved the final version.

For the purposes of open access, the author has applied a Creative Commons Attribution (CC-BY) license to any Accepted Author Manuscript version arising from this submission.

### Sources of Funding

This project is funded by the National Institute for Health and Care Research (NIHR) under its program Grants for Applied Research (NIHR202339) and is supported by the NIHR Applied Research Collaboration South London at King’s College Hospital National Health Service Foundation Trust. The views expressed are those of the authors and not necessarily those of the NIHR or the Department of Health and Social Care. D. Youkee is funded by a Medical Research Council Clinical Research Training Fellowship: MR/W000903/1. Dr Akinyemi is supported by grants U19AG074865, U19 AG076581, and R01AG072547 from the United States.

Dr Kalansooriya works under the project funded by NIHR under its program Grants for Applied Research (NIHR202339).

### Disclosures

None.

### Supplemental Material

Table S1

References 8–29,31–37,40–49,51–54,58–79,81,83

## Supplementary Material


