# Supplementary material for: Socioeconomic Status and Stroke: A Review of the Latest Evidence on Inequalities and Their Drivers
Source: Stroke. 2024 Dec 19;56(3):794–805. doi: 10.1161/STROKEAHA.124.049474 (PMC11850189; doi:10.1161/STROKEAHA.124.049474)
Supplement: Supplementary file 1 [file str-56-794-s001.pdf]

SUPPLEMENTAL MATERIAL

Table S1. Detailed characteristics of studies cited in the review

Title	Study design	Participant count	Methodology	Dependent variables	Measured variables	Main findings	Limitations
**Sex, Age, and Socioeconomic Differences in Nonfatal Stroke Incidence and Subsequent Major Adverse Outcomes**<br><br>**Akyea et al; 2021 (8)**	Observational, retrospective, population-based cohort	68,877	Identifying a cohort with the first nonfatal stroke record, assessing subsequent major adverse outcomes in patients without prior major adverse events, and conducting a sensitivity analysis on outcomes occurring after 30 days of the index stroke.	- Incidence rate of first nonfatal stroke<br>- Incidence rate of subsequent major adverse outcomes	The measured variables in the study include sex, age, SES, incidence rates (IRs) of stroke, and IR ratios (IRRs) for IMD quintiles.	Higher incidence in women, and the impact of age and socioeconomic status on major adverse outcomes in women post-stroke.	- Case ascertainment reliant on clinical codes<br>- Inaccurate recording may affect estimates<br>- Possibility of overestimation of stroke rates<br>- Exclusion of patients without 12 months of data may impact results<br>- Limited completeness of ethnicity information<br>- Ethnic differences not assessed
**Sex difference in the incidence of stroke and its corresponding influence factors: results from a follow-up 8.4 years of rural China hypertensive prospective cohort study**<br><br>**Wang et al; 2019 (9)**	The study design is a large-scale epidemiological prospective study with a median follow-up of 8.4 years	Total: 5097	The methodology involved analysing data from 5097 hypertensive patients aged 35 years and above using Cox proportional hazard models over a median follow-up period of 8.4 years.	Stroke events (ischaemic stroke, haemorrhagic stroke, unclassified stroke)	Body weight, Height, Body mass index (BMI), Blood pressure (BP), Serum glucose, Total cholesterol, Low-density lipoprotein cholesterol (LDL-C), High-density lipoprotein cholesterol (HDL-C), Triglycerides	- The overall incidence of stroke was higher in men compared to women.<br>- Approximately 25% of the sex difference in stroke incidence could be explained by CVRF and education<br>- The sex difference in haemorrhagic stroke was more pronounced, with 63.89% of the difference being explained by CVRF and education	- Study restricted to a country sample in northeast China, limiting generalization of results<br>- Some minor stroke cases may not have been recorded due to individuals not seeking emergency care, potentially leading to underestimation of stroke cases<br>- Other factors that were not recorded, such as genetic factors, could affect the sex difference in stroke incidence, suggesting the need for further research
**The relationship of income on stroke incidence in Finland and China**<br><br>**Yao et al; 2023 (10)**	Observational, retrospective cohort study	China: 571,843, Finland: 4,046,205	Stroke incidence rates were calculated using the Changde Social Health Insurance Database (China) and the Finnish population register (Finland). Cox regression was used to compare stroke incidence by income quintile	Incidence of first-ever stroke (ischemic and haemorrhagic)	Income, employment status, age, sex, ICD-10 stroke diagnosis codes (I61-I64 for ischemic and haemorrhagic stroke)	Higher income was associated with lower overall and subtype stroke incidence. Income disparity was more pronounced for haemorrhagic stroke. Stroke incidence differences were more significant in men than	Limited generalizability to all of China, data missing for 22% of Chinese participants, and reliance on a 5-year stroke-free period to exclude prior cases in Finland.

						in women in both countries.	
**Education and stroke: evidence from epidemiology and Mendelian randomization study**<br><br>**Xiuyun et al; 2020 (11)**	Observational Mendelian randomization study using<br>The Atherosclerosis Risk in Communities (ARIC) study.	Total: 11,509<br>- Basic education: 2,475<br>- Intermediate education: 4,785<br>- Advanced education: 4,249	The methodology involved using Cox hazard regression models to explore the association between education level and incident stroke, as well as employing Mendelian randomization to estimate the causal relationship between education and different types of strokes.	Dependent variables are incident total stroke and incident ischaemic stroke	Race, Gender, Age, Smoking, Drinking, Family income, Height, Weight, BMI, Blood pressure, Physical activity, Diabetes, Stroke, CHD, HF, Total cholesterol, HDL-c, LDL-c, Triglycerides, Creatine	- Participants with advanced education had a 25% decreased rate of incident total stroke.<br>- Education was likely a negative causal risk factor for ischaemic stroke.<br>- Higher education levels were associated with a decreased rate of total stroke and ischaemic stroke incident, but not haemorrhagic.	- Confounders not measured in the ARIC study<br>- Ascertainment of stroke needs improvement<br>- Limited to European populations
**Independent and combined effect of income and education attainment on the incidence of stroke events**<br><br>**Ma et al; 2023 (12)**	Prospective, cohort study from rural communities in China	Total: 15,913	Baseline survey conducted in 2013, with follow-up until 2018 (median follow-up of 5.5 years). Cox proportional hazard models were used to assess stroke risk in relation to income and education levels.	Stroke events (ischemic and non-ischemic stroke).	Monthly household income per capita (≤999, 1000–1499, ≥1500 RMB), Education levels (0 years, 1–5 years, ≥6 years), BMI, smoking status, hypertension, diabetes, hyperlipidaemia, coronary heart disease (CHD).	Lower income and fewer years of education were associated with higher stroke risk (HR for income: 1.54; HR for education: 1.59). Participants with both low income and higher education had the highest risk (HR: 2.46).	The study sample was limited to rural China, which may limit the generalizability. Stroke subtypes like lacunar strokes were not analysed separately. Biomarkers related to stress (e.g., cortisol) were not included.
**Impact of Multiple Social Determinants of Health on Incident Stroke**<br><br>**Reshetnyak et al; 2020 (13)**	Observational prospective cohort study, multi-site	Total: 27,813	Prospective cohort design with a large sample size, using Cox proportional hazards models to examine the associations between incident stroke and social determinants of health (SDOH). The selection of candidate SDOH was guided by the Healthy People 2020 framework, and sensitivity analyses were conducted to address missing data.	Incident stroke based on expert adjudication after medical record review.	Age, sex, region, self-reported history of heart disease, high blood pressure, dyslipidaemia, diabetes mellitus, atrial fibrillation, left ventricular hypertrophy, medication use (antihypertensives, statins, insulin), functional status, health behaviours (smoking, alcohol use, physical activity, diet adherence), physiological variables (body mass index, hsCRP, urinary albumin-to-creatinine ratio, estimated glomerular filtration rate)	- The study observed 1470 incident strokes over a median follow-up of 9.5 years.<br>- Among adults aged <75 years, the incidence of stroke increased as the number SDOH within the same individual increased, with the risk nearly two and a half times higher in those with ≥3 SDOH compared to those with no SDOH.<br>- After adjusting for potential confounders, stroke risk remained 50% higher among	- Self-reported variables may introduce recall bias<br>- Limited information on tobacco exposure beyond cigarette smoking<br>- Lack of data on additional SDOH like perceived discrimination or environmental factors<br>- Potential missed stroke events due to reliance on reaching participants

						individuals with ≥3 SDOH compared to those without any SDOH.	
**Occupational Class and Risk of Cardiovascular Disease Incidence in Japan: Nationwide, Multicenter, Hospital-Based Case-Control Study**<br><br>**Zaitsu et al; 2019 (14)**	Observational, retrospective, matched hospital case-control study with a large sample size of over 1.1 million study subjects.	The participant count is 1,128,591.	The methodology involved using a nationwide hospital inpatient dataset, classifying patients by their longest-held occupational class, conducting conditional logistic regression with multiple imputation, and controlling for smoking and drinking.	Coronary heart disease (CHD) incidence, Stroke incidence, Overall cardiovascular disease (CVD) incidence	Longest-held occupational class (blue-collar, service, professional, manager) and industrial sectors (blue-collar, service, white-collar)	- In Japan, managers and professionals may have a higher risk for coronary heart disease.<br>- The typical inverse socioeconomic gradient in cardiovascular disease may not be universal.<br>- There are opposite socioeconomic gradients for coronary heart disease and stroke in Japan.	- Selection of hospital controls potentially subject to bias<br>- Residual statistical differences in baseline characteristics between cases and controls due to the matching procedure<br>- Relevant socioeconomic factors like educational attainment and income were not assessed<br>- Inability to assess severity of disease at admission, other conventional risk factors, and workplace-related risk factors
**Effect of income level on stroke incidence and the mediated effect of simultaneous diagnosis of metabolic syndrome diseases; a nationwide cohort study in South Korea**<br><br>**Jeong et al; 2022 (15)**	Retrospective cohort study based on the National Health Insurance Service National Sample Cohort (NHIS-NSC)	Total: 213,526	Analysis was conducted using multivariate logistic regression and Cox proportional hazards model. Income level was classified into high, middle, low, and Medical Aid beneficiaries. A causal mediation analysis was employed to examine the mediation effects of metabolic syndrome (MetS) components.	Stroke incidence	Income level, simultaneous diagnosis of MetS components (hypertension, diabetes mellitus, dyslipidaemia), age, sex, comorbidities (CCI), residence, disability	- Low-income groups and Medical Aid beneficiaries had a significantly higher risk of stroke compared to the high-income group.<br>- Co-diagnosis of MetS components mediated 26.6% (for two or more MetS components) and 21.1% (for three MetS components) of the stroke risk in the Medical Aid group.	- Income level was assumed to remain unchanged over the observation period, potentially introducing bias.<br>- Diagnosis of MetS components was based on medical records, which may differ from the actual prevalence.<br>- Did not account for the duration of MetS diseases or unmeasured confounders like pharmacological treatments.
**Neighbourhood Socioeconomic Status at the Age of 40 Years and Ischaemic Stroke before the Age of 50 Years -a Nationwide Cohort Study from Sweden**	Observational, retrospective, population-based, non-randomised, non-controlled	1,153,451 adults (women: 48.9%)	The methodology involved using national registers to categorize neighbourhood SES, tracking individuals from a specific age range, utilizing small area market statistics for neighbourhood proxies, and employing Cox regression models to estimate hazard	Incident ischaemic strokes among individuals below 50 years of age	Marital status, Education level, Immigrant status, Region of residence	Neighbourhoods with low SES were associated with a higher risk of stroke, while neighbourhoods with high SES were associated with a lower risk of stroke compared to neighbourhoods with middle socio-economic	- Lack of data on established CVRF<br>- Results may not be generalisable to other age-groups<br>- Lack of information on individual-level cardiovascular risk factors

**Carlsson et al; 2017 (16)**			ratios and confidence intervals.			status. The relationship remained significant after adjusting CVRF and no significant interaction with sex was found.	- Potential limitations in generalizability beyond Sweden<br>- Lack of detailed information on specific comorbidities<br>- Lack of information on lifestyle factors that could impact stroke risk
**Causal nature of neighbourhood deprivation on individual risk of coronary heart disease or ischaemic stroke: a prospective national Swedish co-relative control study in men and women**<br><br>**Forsberg et al; 2018 (17)**	Observational study with a co-relative design	Men: 23,479, 66,978 and 72,257 individuals per age group.<br>Women: 10,383, 30,332 and 42,607 respectively	The methodology involved using nationwide Swedish registers, Cox proportional hazards models, stratified Cox regression models.	- Incidence of coronary heart disease<br>- Incidence of ischaemic stroke	Family income, educational attainment, Neighbourhood SES	Neighbourhood SES was associated with incidence of CHD and ischaemic stroke, and the present study suggests that these associations were, at least in part, causal.	- The study is limited to Sweden<br>- Residual confounding may exist<br>- Family-based designs do not eliminate issues of residual confounding<br>- Exclusion of individuals who had lived abroad<br>- Possible limitations in using socioeconomic measures as proxies for individual-level status
**Associations between socioeconomic status and stroke in American adults: A population-based study**<br><br>**Fan et al, 2023 (18)**	Observational study utilizing data from the NHANES database between 2009 and 2018, employing a stratified, multistage probability design to provide a representative sample of the US population. The study is retrospective, cross-sectional, and observational in nature.	22,792	The methodology involved collecting data from the NHANES database, conducting weighted multivariate logistic regression analysis and subgroup analysis, using statistical tests for data analysis, and employing R software for statistical analysis.	Stroke incidence, educational level, and the relationship between poverty to income ratio (PIR) and stroke incidence.	Poverty to income ratio (PIR), Education level, Age, Sex, Race/ethnicity, Coronary heart disease, Hypertension, Hyperlipidaemia, Diabetes, Body mass index (BMI)	- Poverty to income ratio (PIR) is a protective factor for stroke<br>- The protective effect of PIR against stroke becomes stronger as the PIR level increases.	- Multiple-subject design creating challenges in establishing causality<br>- Potential influence of residual and unmeasured confounders

**Education, sex and risk of stroke: a prospective cohort study in New South Wales, Australia**<br><br>**Jackson et al; 2018 (19)**	Prospective cohort study conducted in New South Wales, Australia,	Total: 253,657	Cox regression was used to calculate hazard ratios, and adjustments were made for various factors. Data imputation was performed for missing values.	First-ever stroke events.	Education level, marital status, geographical remoteness, area-based deprivation, and average household income.	Low education is associated with increased stroke risk in both men and women, with a slightly stronger association in women. Modifiable lifestyle factors explain much of the excess risk, and the association weakens in older age groups.	- Inability to identify non-fatal strokes not admitted to hospital<br>- misclassification of stroke diagnosis<br>- potential overadjustment in analyses
**Longitudinal Associations Between Income Changes and Incident Cardiovascular Disease The Atherosclerosis Risk in Communities Study**<br><br>**Wang et al; 2019 (20)**	The study design is a prospective, observational cohort study.	The total participant count is 8,989.	1. Use of data from the ARIC prospective cohort study<br>2. Categorization of participants based on changes in household income over 6 years<br>3. Examination of the incidence of cardiovascular disease over 17 years of follow-up<br>4. Adjustment for various sociodemographic, behavioural, and biomarker factors at baseline	- Incident cardiovascular disease (CVD), including myocardial infarction (MI), fatal coronary heart disease (CHD), heart failure (HF), and stroke<br>- Definite or probable MI or fatal CHD<br>- Incident HF<br>- Definite or probable stroke	- Household income (at visit 1)<br>- Sociodemographic variables (age, sex, race/ethnicity, education, employment, household size, insurance status)<br>- Health behaviours (smoking, alcohol use, physical activity)<br>- Biomarkers (BMI, cholesterol, blood pressure, glucose, creatinine)	- Income drop over 6 years was associated with a 17% higher risk of subsequent incident cardiovascular disease over 17 years of follow-up.<br>- Income rise over 6 years was associated with a 14% lower risk of subsequent incident cardiovascular disease over 17 years of follow-up.<br>- The association between income drop and higher cardiovascular disease risk was mainly driven by higher risk of myocardial infarction/fatal coronary heart disease and stroke.	- No standard definition of "significant" income change<br>- Potential misclassification of income changes over time<br>- Possibility of reverse causation (i.e. poor health leading to income loss)<br>- Potential effects of long-term income levels rather than just changes<br>- Unclear reasons for income changes beyond just the magnitude
**Neighbourhood socioeconomic index and stroke incidence in a national cohort of blacks and whites**<br><br>**Howard et al; 2016 (21)**	Observational, retrospective	Black (n 5 10,274, 41%) and white (n 5 14,601) stroke-free participants	Patients enrolled in 2003–2007 in REGARDS, a national population-based cohort. A neighbourhood socioeconomic score was constructed Incident stroke was defined as first occurrence of stroke. Proportional hazards models were used to estimate associations	Stroke incidence	Neighbourhood SES score and incident stroke, demographics (age, race, sex, region), individual SES: (education, household income), and other risk factors for stroke.	Compared to the highest neighbourhood quartile, stroke incidence increased with each decreasing quartile. T<br>After adjustment for individual SES, the trend remained marginally significant (p 5 0.085).<br>No evidence of a differential effect by race or sex, adjustment for stroke risk factors attenuated the	Those who agreed to participate in REGARDS may not be representative of the general population, potentially reducing generalisability.<br><br>By design, the study only included black and white participants, so the diversity of racial/ethnic groups in the United States is not represented.

						association between neighbourhood and stroke, with greater attenuation in black participants.	
**Race and Ethnic Disparities in Stroke Incidence in the Northern Manhattan Study**<br><br>**Gardener et al; 2020 (22)**	Prospective population-based cohort study with observational design and multivariable-adjusted Cox proportional hazards models	3298	The methodology involved using Cox proportional hazards models to analyse the association between race/ethnicity and incident stroke, stratified by age, in a population-based study with a focus on vascular risk factors and self-identified race/ethnicity categories.	Incident cases of any stroke type (infarcts, intracerebral haemorrhage, and subarachnoid haemorrhage) and ischaemic stroke subtypes (lacunar infarct, extracranial atherosclerotic infarct, intracranial atherosclerotic infarct, cardioembolic infarct, and cryptogenic infarct/conflicting mechanisms).	Medical history (hypertension, diabetes mellitus, hypercholesterolemia), medication use, health behaviours (cigarette smoking, alcohol intake), blood pressure, blood glucose, lipid levels, body mass index, proxies for SES neurological symptoms, events/diagnoses, hospitalizations, death	The study identified disparities in stroke risk among different racial and ethnic groups, with higher incidence rates observed in blacks compared to whites, and the increased risk among Hispanics being partially explained by SES. Disparities were more significant in women, particularly in the older age group.	- Lack of power to detect significant race/ethnic disparities in stroke risk over multiple strata<br>- Insufficient power to estimate race/ethnic disparities in ischaemic stroke subtypes and haemorrhagic stroke subtypes, especially by age category
**Impact of Neighbourhood Socioeconomic Conditions on the Risk of Stroke in Japan**<br><br>**Honjo et al; 2015 (23)**	Prospective cohort study, observational.	90,843	Association between neighbourhood deprivation and the risk of stroke in Japan, adjusting for various factors such as individual socioeconomic conditions and biological cardiovascular risk factors.	Stroke mortality, Stroke incidence	Occupation, age, gender, district, population density, biological cardiovascular risk factors, and behavioural/psychosocial factors	- Neighbourhood deprivation level was significantly associated with the risk of stroke incidence in Japan.<br>- The impact of neighbourhood deprivation on stroke risk remained significant even after adjusting for individual SES	- Limited generalisability of study results due to the inclusion of only nine public health districts in a non-metropolitan setting<br>- 5-year gap between census data and baseline data could impact the results<br>- Adjustment for individual-level SES using occupation only may not be sufficient
**Sex differences in the association between major risk factors and the risk of stroke in the UK Biobank cohort study**<br><br>**Peters et al; 2020 (24)**	Observational cohort study	Participant count: 471,971	The methodology involved using data from the UK Biobank, obtaining informed consent, collecting detailed participant information, and adjudicating incident stroke cases through hospital data and death registers.	Incidence of fatal or nonfatal ischaemic stroke, haemorrhagic stroke, and all strokes; Relationship between blood pressure variables and the risk of any stroke and stroke subtypes; Association of hypertension, smoking, type 1 diabetes, body anthropometry, lipid fractions, atrial fibrillation, and socioeconomic status with the risk of stroke in both women and men.	Blood pressure (SBP, DBP), pulse pressure, mean arterial pressure, hypertension categories, body weight, waist and hip circumference, standing height, BMI, overweight and obesity status, waist-to-hip ratio, waist-to-height ratio, blood lipid levels (total cholesterol), socioeconomic status	The main findings of the study include that several risk factors such as hypertension, smoking, and low SES were more strongly associated with the risk of stroke in women compared to men.	- Self-reported information on key variables could introduce measurement error<br>- Possibility of residual confounding despite adjusting for several confounders<br>- Limited generalisability to other ethnicities due to most participants being of White ancestry

**Determinants of social inequalities in stroke incidence across Europe**<br><br>**Ferrario et al; 2017 (25)**	Observational, prospective cohort study	Total: 126,635	The MORGAM Study comprises 48 cohorts from 4 European regions, standardised procedures for risk factor assessment, and categorisation of educational levels based on years of schooling.	Stroke incidence rates, hazard ratios for stroke incidence, educational class inequalities in stroke incidence, and risk factors contributing to stroke incidence inequalities.	The measured variables in the study are blood pressure, total cholesterol, HDL-cholesterol, Body Mass Index (BMI), cigarette smoking, alcohol intake, and history of diabetes.	- Educational class inequalities significantly impact stroke incidence rates, with the least educated individuals facing a higher risk compared to the most educated.<br>- Inequalities in risk factors, particularly related to behaviours like smoking, alcohol intake, and body mass index, play a substantial role in determining the social gap in stroke incidence, especially in certain European regions.	- Risk factors were measured only once at baseline, potentially leading to residual confounding.<br>- Alcohol intake assessment was based on average daily consumption without considering binge drinking.<br>- Some centres had a small number of events, affecting the stability of estimates.<br>- Participation rates were below 60% in some populations, possibly introducing selection bias.<br>- Lack of data on overall caloric intake, usual diets, physical activity levels, and stress-related factors may have underestimated the contribution of behavioural risk factors.
**Combined effect of educational status and cardiovascular risk factors on the incidence of coronary heart disease and stroke in European cohorts: Implications for prevention**<br><br>**Veronesi et al; 2016 (26)**	The study design is a prospective cohort study.	77,918 participants	- Prospective cohort study of 77,918 individuals aged 35-74 from 38 cohorts across Europe, followed for a median of 12 years<br>- Used Fine-Gray competing risk models to estimate the interaction of education with smoking, blood pressure, and BMI on the cumulative risk of CHD and stroke<br>- Collected education level via a standardized questionnaire item on years of schooling<br>- Measured cardiovascular risk factors at baseline using MONICA protocols<br>- Ascertained CHD and stroke events through linkage to national/regional registries and direct participant follow-up	- Cumulative incidence of coronary heart disease (CHD)<br>- Cumulative incidence of stroke<br>- Composite endpoint of first CHD or stroke	- Total cholesterol<br>- HDL-cholesterol<br>- Blood pressure<br>- BMI<br>- Smoking status	- Low educational status was associated with an increased cumulative risk of coronary heart disease and stroke in both men and women.<br>- The interaction between low education and smoking led to an added increase in the cumulative risk of cardiovascular disease events.<br>- The interaction between low education and obesity/overweight was negative, suggesting that more educated individuals would benefit more from weight reduction.	factors on the incidence of coronary heart disease and stroke in European cohorts: Implications for prevention Giovanni Veronesi +17 European Journal of Preventive Cardiology veronesi2016.pdf 2017 ·<br>49 citations<br>- The data is from the 1980s and 1990s, so may not reflect more recent trends in social inequalities and risk factors.<br>- Risk factors were only measured at baseline, so changes over time (e.g. smoking cessation) may have been missed.<br>- Participation rates were low in some studies, which could

							affect estimates of inequalities.<br>- The study did not aim to explain the causal mechanisms behind the observed interactions.
**Socioeconomic disparities in first stroke incidence, quality of care, and survival: a nationwide registry-based cohort study of 44 million adults in England**<br><br>**Bray et al, 2018 (27)**	Observational cohort study	Total: 145,324<br>- Ischaemic stroke: 126,640<br>- Intracerebral haemorrhage: 17,233<br>- Undetermined stroke type: 1451	Socioeconomic status was classified at the level of Lower Super Output Areas using the Index of Multiple Deprivation. Multivariable models were used to estimate the incidence of hospitalization, quality of care, and 1-year case fatality. Data from the Sentinel Stroke National Audit Programme (SSNAP) was utilized for the study. The analysis included negative binomial regression, multilevel logistic regression, and Cox proportional hazards models.	- Incidence of hospitalisation for first stroke<br>- Quality of care using 12 quality metrics<br>- All-cause 1 year case fatality	Quality of care metrics from the SSNAP data	- There is a socioeconomic gradient in the hospitalization incidence for both ischaemic stroke and intracerebral haemorrhage, with higher rates observed in the most deprived deciles.<br>- Low socioeconomic status is associated with a higher risk of 1-year mortality after stroke, but this association is partially explained by pre-stroke cardiovascular risk factors.<br>- Disparities exist in certain aspects of acute stroke care, such as anticoagulation for atrial fibrillation and timely admission to a specialist stroke unit.	- Area-based measure of socioeconomic status used instead of person-level measures<br>- Lack of information on important stroke risk factors like smoking status, body-mass index, or physical activity<br>- Limited outcome data focusing only on mortality<br>- Quality metrics mainly related to acute stroke care, lacking metrics on community-based or follow-up services<br>- Insufficient data on ethnicity profile of Lower Super Output Areas (LSOAs)<br>- Case ascertainment through routine care may be less complete compared to an ideal population-based register<br>- Exclusion of patients not admitted to the hospital or with transient ischaemic attack may lead to underestimation of the total population burden of stroke

**The effect of socioeconomic status on three-year mortality after first-ever ischaemic stroke in Nanjing, China**<br><br>**Zhou et al; 2020 (28)**	Observational study, retrospective	806	The methodology involved enrolling 806 patients with first-ever ischaemic stroke and using SES indicators along with stepwise univariate and multivariate COX proportional hazards models to study the association with three-year mortality.	- Occupation<br>- Taxable income<br>- Housing space	Level of education, occupation, taxable income, housing space	Lower SES indicators such as occupation, income, and housing space were significantly associated with three-year mortality after first-ever stroke. Manual workers, individuals with zero income, and those with smaller housing spaces had significantly higher hazard ratios for death within three years compared to their counterparts.	- Hospital-based study may introduce selection bias<br>- Results may not be generalisable to the overall Chinese population due to geographical differences<br>- Elderly patients with minor strokes may not have been hospitalized<br>- Stroke patients in rural areas may have been misdiagnosed or not treated according to modern standards<br>- Cultural differences may have influenced seeking medical attention and enrolment in the stroke registry
**Gender difference of geographic distribution of the stroke incidence affected by socioeconomic, clinical and urban-rural factors: an ecological study based on data from the Brest stroke registry in France**<br><br>**Padilla et al; 2021 (29)**	Ecological study based on the Brest Stroke Registry, investigating stroke cases in individuals aged 60 years or older between 2008 and 2013,	Total: 3088<br>- Women: 1743<br>- Men: 1345	The methodology involved extracting stroke cases aged 60 years or more from the Brest stroke registry, utilizing contextual variables from the 2013 national census, and employing spatial and non-spatial regression models to study the relationship between socioeconomic deprivation, urbanization level, and stroke incidence. Maps were generated using spatial geographically weighted models.	- Stroke incidence in women<br>- Stroke incidence in men	sociodemographic data (age and sex); clinical data, including stroke type (ischaemic or haemorrhagic), stroke severity (National Institute of Health Stroke Score (NIHSS) score < 6, 6-13, > 13); presence of cardiovascular risk factors before stroke (high blood pressure, cardiac arrhythmia, diabetes, and dyslipidaemia)	The study found different patterns of stroke risk distribution and its association with deprivation and urbanization across gender, with women showing homogeneous risk associated with deprivation in urban areas, and men having three clusters with varying deprivation and urbanization levels affecting stroke risk.	- Coverage of the registry is around 80%<br>- Multiple imputation was used to account for missing information
**Impact of armed conflict on cardiovascular disease risk: a systematic review**<br><br>**Jawad et al; 2019 (31)**	Systematic review	Participant count is not directly mentioned in the paper. The study included 65 studies analysing 23 armed conflicts, but the specific number of participants randomized into each trial arm is not provided.	Systematic review of multiple databases without restrictions on language or date, inclusion of adult civilian populations in LMICs, reanalysis of data descriptively, recalculating data from different study designs, summarising effect directions and study quality, reporting outcomes by ICD-10 codes using harvest plots, and visually assessing publication bias with an adapted funnel plot.	- Cardiovascular diseases (CVDs)<br>- Diabetes<br>- Blood pressure<br>- Blood glucose<br>- Lipids<br>- Tobacco use<br>- Alcohol use<br>- Body mass index<br>- Nutrition<br>- Physical activity	Cardiovascular diseases (CVD), diabetes, blood pressure, blood glucose, lipids, tobacco use, alcohol use, body mass index (BMI), diet, physical activity	The main findings highlight the association between armed conflict and increased cardiovascular disease risk factors and stroke	Limited number of studies, mainly of low quality; inability to formally assess publication bias; majority of evidence from a few conflicts; use of scoring for risk of bias assessments
**Is War Hard on the Heart? Gender, Wartime Stress and Late Life Cardiovascular**	Observational, retrospective	3,696	Answer not found	- Hypertension<br>- Dyslipidaemia<br>- Heart disease<br>- Stroke	Hypertension, dyslipidaemia, heart disease, stroke, blood pressure measurements, self-reported diagnosis of cardiovascular conditions,	The main findings highlight the enduring influence of early life wartime stressors on stroke and	Limitations associated with self-reports of health conditions, potential recall bias in measuring early life

**Conditions in a Population of Vietnamese Older Adults**<br><br>**Korinek et al; 2020 (32)**					demographic, and lifestyle variables	cardiovascular disease in late adulthood, with PTSD mediating this association and Vietnamese women experiencing a significant stroke burden alongside men.	wartime stressors, and timing ambiguity in assessing life event stressors and their relation to CVD condition onset.
**Incidence of stroke, transient ischaemic attack and determinants of poststroke mortality among immigrants in Denmark, 2004-2018: a populationbased cohort study**<br><br>**Mkoma et al; 2021 (33)**	Population-based cohort study between 2004 and 2018, utilizing registry data and Cox proportional hazard regression.	132,936	Quantifying the incidence of stroke and TIA among immigrants compared to Danish-born individuals using registry data, estimating age-standardised incidence rate ratios, and using Cox proportional hazard regression to calculate the risk of poststroke mortality. Data from Danish nationwide registers were utilized.	Stroke (including IS, ICH, and unspecified stroke), TIA, Poststroke mortality	Age at stroke, Age at immigration, Sex, Smoking, Comorbidities, Stroke severity, Thrombolytic or endovascular therapy, Education, Employment status, Income, Marital status, Duration of residence	Immigrants, both Western (German, Norwegian, Swedish and Polish) and non-Western (Turkish, Polish, German, Norwegian, Iraqi, Swedish, Pakistani, Ex-Yugoslavian and Iranian. Furthermore, Turkish, Iraqi, Pakistani, Ex-Yugoslavian and Iranian), had a higher risk of stroke and TIA compared to Danish-born individuals.	- Lack of individual-level data on comorbidities and SES<br>- Not considering the impact of various lifestyle factors on poststroke mortality<br>- Need for further research due to immigrants' exposure to deprived social conditions
**Processes of Care and Associated Factors in Patients With Stroke by Immigration Status**<br><br>**Mkoma et al; 2023 (34)**	The study design is an observational nationwide register-based cohort study.	Total: 129,724<br>- Danish-born residents: 123,928<br>- Immigrants: 5,796	The methodology involved a nationwide register-based cohort study using data from the Danish Stroke Registry, multivariable logistic regression models, composite scores to evaluate stroke care quality, subgroup analyses, and interaction term assessments.	The dependent variables in the study are the early stroke care processes, including early stroke unit admission, early dysphagia screening, early physiotherapy, early occupational therapy, and nutritional assessment.	Immigration status, region of origin, country of origin, in-hospital processes of stroke care, opportunity-based composite score, all-or-none composite score	Immigrants had lower chances of early stroke unit admission and received fewer individual stroke care processes compared to Danish-born residents, with disparities more pronounced for non-western immigrants. Overall disparities in stroke care were minor and largely explained by socioeconomic factors.	Rely on professional medical interpreters in the quality of acute stroke care delivery as such data was unavailable.<br><br>The proportion of immigrants was increasing over time and not enough in the early part of the study period to explore the temporal trends in stroke care processes.
**Use of reperfusion therapy and time delay in patients with ischaemic stroke by immigration status: A register-based cohort study in Denmark**<br><br>**Mkoma et al; 2022 (35)**	The study design is a nationwide register-based cohort study, which is a retrospective observational study using nationwide registry data.	The total number of participants in the study was 49,817, with 2,083 immigrants and 47,734 Danish-born residents.	- Nationwide register-based cohort study of patients with acute ischemic stroke aged ≥18 years (n = 49,817) from 2009 to 2018<br>- Data sources: Danish Stroke Registry, Danish Prehospital Database, and Statistics Denmark<br>- Immigrants classified by country of origin (Western, non-Western, Turkey, Poland, Pakistan)<br>- Outcomes: use of reperfusion therapy, time delays (onset-to-call, onset-to-door, door-to-needle), and functional outcome	- Use of reperfusion therapy (IV thrombolysis and/or mechanical thrombectomy)<br>- Time delays: onset-to-call time, onset-to-door time, and door-to-needle time<br>- Functional outcome	- Reperfusion therapy (intravenous thrombolysis and/or mechanical thrombectomy)<br>- Time delays (onset-to-call time, onset-to-door time, door-to-needle time)<br>- Stroke severity (as measured by the Scandinavian Stroke Scale)<br>- Duration of residence in Denmark (both as a continuous variable and a categorical variable)	- Immigrants had 33% lower chances of receiving reperfusion therapy compared to Danish-born residents, after adjusting for relevant confounders.<br>- Immigrants had a 15-minute longer prehospital delay compared to Danish-born residents among patients arriving within 4.5 hours of stroke onset, after adjusting for relevant factors.<br>- No differences were found in system-related	- Observational nature, so only associations can be explored, not causation<br>- Possibility of random variation due to small sample size<br>- Limited number of immigrant stroke patients, requiring caution in interpreting country-specific analyses<br>- Lack of detailed information on reasons for reperfusion therapy contraindications, and exclusion of 20% of

						delays or clinical outcomes between immigrants and Danish-born residents among patients eligible for reperfusion therapy.	the stroke population due to missing onset-to-door time data
**Incidence of Stroke in Immigrants to Canada A Province-wide Retrospective Analysis**<br><br>**Vyas et al; 2021 (36)**	Retrospective cohort study, observational design	8,090,524	Adults in Ontario, Canada, from 2003 to 2018, using cause-specific hazard models to calculate adjusted hazard ratios and evaluating the association by age, stroke type, and country of origin of immigrants.	Incidence rate of stroke or TIA events, the hazard ratio of stroke or TIA in immigrants compared to long-term residents, the crude incidence rate and hazard of stroke or TIA in immigrants compared to long-term residents, and the reduction in adjusted hazard in immigrants for different stroke types.	Age, sex, neighbourhood-level income, comorbid conditions (hypertension, diabetes, dyslipidaemia, atrial fibrillation, COPD, congestive heart failure), information on immigrants arriving in Ontario	Immigrants in Ontario had a 33% lower rate of stroke or TIA compared to long-term residents, with consistency across different stroke types and age groups. The reduction in stroke risk associated with immigration status was less pronounced in immigrants from the Caribbean, Latin America, and Africa compared to those from other world regions.	- The study did not have information on potential risk factors such as education, family history, smoking, alcohol, diet, physical activity, or body mass index.<br>- The study did not have information on screening or control of vascular risk factors such as hypertension.<br>- The study did not have information on the classification of stroke type or its aetiology.<br>- The study was not able to identify unregistered immigrants, potentially leading to a bias as this group may be less likely to seek medical care.
**Immigration Status, Ethnicity, and Long-term Outcomes Following Ischaemic Stroke**<br><br>**Vyas et al; 2021 (37)**	Retrospective cohort study using linked administrative and clinical registry-based data, with analysis stratified by age and ethnicity, and using inverse probability of treatment weighting.	31,918	Hazards of all-cause mortality and vascular event recurrence comparison in immigrants and long-term residents using inverse probability of treatment weighting accounting for age, sex, income, and comorbidities.	Mortality and vascular event recurrence	Ethnicity, clinical information, baseline characteristics, vascular risk factors, medical comorbidities, treatment, outcomes	- Immigrants had lower mortality rates, especially among those younger than 75 years.<br>- Mortality varied by ethnicity, with higher rates in South Asian immigrants, similar rates in Chinese immigrants, and lower rates in other immigrants.<br>- Recurrence risk was similar between immigrants and long-term residents.	- The study lacked data on important risk factors such as diet, physical activity, and obesity.<br>- The study did not have information on the severity and duration of known risk factors like diabetes, hypertension, and hyperlipidaemia.<br>- The age distribution of immigrants included in the long-term resident category was not determined.<br>- Stroke incidence in different immigrant groups was not determined, which is crucial for understanding overall stroke mortality rates.

**Synergism of Short-Term Air Pollution Exposures and Neighbourhood Disadvantage on Initial Stroke Severity**<br><br>**Wing et al; 2017 (40)**	Observational, retrospective study	3035	Identifying ischaemic stroke cases, determining stroke severity, and modelling the associations between neighbourhood disadvantage, air pollutants, and stroke severity using regression analysis.	- Initial stroke severity measured by the National Institutes of Health Stroke Scale (NIHSS) score<br>- Odds of severe stroke (defined as NIHSS score ≥7)	Same-day PM 2.5 and previous-day O3 concentrations	- Neighbourhood disadvantage and short-term exposure to air pollutants were associated with higher odds of severe stroke.<br>- The association between particulate matter and severe stroke was dependent on neighbourhood disadvantage levels.<br>- Higher ozone levels were consistently associated with stroke severity.	- Use of only one air pollution monitor<br>- Potential inaccuracies in capturing neighbourhood exposures with census tracts<br>- Differences in seeking medical treatment between lower and higher SES areas
**Air pollution and stroke; effect modification by sociodemographic and environmental factors. A cohort study from Denmark**<br><br>**Poulsen et al; 2023 (41)**	Observational cohort study	1,964,702	Assessing long-term exposure to air pollution, calculating correlations, considering potential confounders, establishing comorbidity indices, quantifying green space and road traffic noise, and using Cox and Aalen models to estimate associations between air pollution and stroke risk.	- Incident stroke cases identified during the follow-up period<br>- Long-term exposure to air pollution with ultrafine particles, PM 2.5, elemental carbon, and NO2<br>- Associations between air pollution and stroke, with a focus on individuals with comorbidities, shorter education, lower income, and being retired<br>- Stronger associations observed among individuals living in less populated areas, with low noise levels, and more green space around the residence	PM 2.5 concentrations, EC concentrations, NO2 concentrations, UFP concentrations, financial stress events, Charlson comorbidity index, population density, proportion of parish inhabitants with only mandatory education, green space within 150 m of the residence, road traffic noise levels	The study did not account for cardiovascular risk factors, strokes subtypes were not individually analysed	-
**Long-Term Exposure to Transportation Noise and Risk of Incident Stroke: A Pooled Study of Nine Scandinavian Cohorts**<br><br>**Roswall et al; 2021 (42)**	Observational study	11,056	Pooled data from nine Scandinavian cohorts, estimating noise exposure for each address, linking to national registries for stroke data, and using Cox proportional hazards models for analysis.	incident stroke.	PM 2.5 concentrations, covariates selected a priori	Road traffic noise was associated with an increased risk of stroke, while moderate levels of aircraft noise were also linked to stroke incidence. Railway noise did not show an association with stroke. These associations varied after the adjustments of educational level	Cohort-specific estimation of noise, which did not follow a standardised protocol
**Transportation noise exposure and cardiovascular mortality: a nationwide cohort**	Observational cohort study with longitudinal design and exposure assessment using a novel noise metric.	Total: 4.41 million	Generating exposure models for transportation noise sources in Switzerland for 2001 and analysing their association with	- Primary causes of death from all cardiovascular diseases (CVD)<br>- Ischaemic heart disease (IHD)	Exposure to road traffic noise, railway noise, aircraft noise (L den above 45 dB), and night-time intermittency ratio (IR)	The main findings highlight the impact of major transportation noise sources on cardiovascular diseases, with a focus on	- Potential for exposure misclassification due to modelling errors and missing data<br>- Uncertainty from missing information

**study from Switzerland**<br><br>**Héritier et al 2017 (43)**	controlled, or blinded.		cardiovascular mortality using Cox regression models.	- Stroke in general<br>- Haemorrhagic stroke<br>- Ischaemic stroke<br>- Myocardial infarction (MI)<br>- Heart failure<br>- Blood-pressure related death		myocardial infarction and the significance of the intermittency ratio at night in a bell-shaped relationship with cardiovascular diseases.	about residential history and personal behaviour<br>- Errors in the propagation models could lead to underestimation of risks<br>- Lack of information on individual lifestyle and smoking in the Swiss National Cohort
**Effect of Socioeconomic Status and Underlying Disease on the Association between Ambient Temperature and Ischaemic Stroke**<br><br>**Cho et al; 2018 (44)**	Observational study	63564 ED visits	The dependent variables in the study are the number of emergency department (ED) visits for ischaemic stroke, relative risks (RR) and 95% confidence intervals (CI) per a 1°C increase in temperature above/below the thresholds, optimum ambient temperature thresholds in summer and winter, and the risk of ED visits for ischaemic stroke related to temperature.	- Patients who visited the emergency department for ischaemic stroke in Seoul, Korea between 2005-2009<br>- Patients aged 40 years and older<br>- Patients with underlying diseases such as cardiovascular disease and diabetes mellitus<br>- Specifically, patients with diabetes showed increased risk of ED visits for ischaemic stroke with temperature increases above 25.8°C<br>- Elderly patients and medical aid beneficiaries were more vulnerable to ischaemic stroke as temperature decreased in winter	ED visits for ischaemic stroke, Age, Gender, National health insurance type, Date of the visit, Diagnosis code based on the ICD-10 system, Cardiovascular diseases, Diabetes mellitus, Daily average temperature, Relative humidity, Holidays, Day of the week, Air pollutants (PM10 and O3)	- In summer, there was a positive association between ambient temperature increases above a certain threshold and ED visits for ischaemic stroke in patients with diabetes and medical aid beneficiaries.<br>- In winter, there was an inverse association between temperature and ischaemic stroke visits, with the risk significantly increasing as the temperature decreased above a specific threshold.	- Diagnosis of ischaemic stroke based on ED visits without confirmation by neurologists or imaging studies<br>- Hospitalization cases had higher specificity but might not capture all cases due to potential financial barriers<br>- Single-city study limits generalizability to other geographic areas<br>- Hospitalization as an outcome may not fully address socioeconomic inequality due to potential exclusion of patients who cannot afford hospital admission
**Association between ambient temperature and risk of stroke morbidity and mortality: A systematic review and meta-analysis**<br><br>**Wen et al; 2023 (45)**	Systematic review and meta-analysis,	20 studies included	The methodology involved a systematic search of databases, definition of heat and cold ambient temperature, calculation of pooled estimates using a random-effects model, and data extraction based on specific inclusion criteria.	Stroke morbidity, Stroke mortality	Country, study design, sample size, exposure, temperature range, outcome, model, lag days, population, effect indicator (hazard ratio, RR, or odds ratios [OR] with 95% confidence interval [CI]), controlled variables (humidity, air pollution, holiday, day of week and season), quality assessment	Heat and cold ambient temperatures were significantly associated with increased risk of stroke morbidity and mortality. Heat was linked to a 10% increase in morbidity and a 9% increase in mortality, while cold was associated with a 33% increase in morbidity and an 18% increase in mortality.	- Inclusion of both time-series and case-crossover studies<br>- Limitations in proving causation of disease<br>- High degree of heterogeneity between included studies<br>- Need for future multicentre, long-term longitudinal studies<br>- Need for investigations on more precise temperature measurement standards or reference values<br>- Worthwhile research in countries of different

							socioeconomic status, especially in developing countries
**Socioeconomic status and survival after stroke using mediation and sensitivity analyses to assess the effect of stroke severity and unmeasured confounding**<br><br>**Lindmark et al; 2020 (46)**	Observational study, using individual patient data from Riksstroke	Participant count:<br>- Total: 57,936<br>- Ischaemic stroke: 51,159<br>- Intracerebral haemorrhage: 6,777	The methodology involved using causal mediation analysis to decompose the effect of low income on 3-month case fatality into direct and indirect effects due to stroke severity, with sensitivity analysis to assess robustness to unobserved confounding.	Death 0-3 months after stroke, level of consciousness on admission to the hospital, and stroke severity.	Patient age, sex, living arrangements, cardiovascular risk factors (atrial fibrillation, diabetes, smoking history), and stroke types (intracerebral haemorrhage and ischaemic stroke)	- Low SES is associated with increased risk of death after stroke, even with universal healthcare access.<br>- Half of income-related inequalities in stroke fatality are mediated through differences in stroke severity.<br>- Low-income patients have an increased risk of death within 3 months after stroke compared to mid/high income patients.	- The study did not adjust for other SES variables, such as education level, which could have provided a more comprehensive understanding of the impact of SES on survival after stroke.<br>- The study made assumptions about the directions of associations between variables, which may introduce bias or oversimplification in the analysis.
**Social inequalities and gender differences in healthcare management of acute ischaemic stroke in France**<br><br>**Naouri et al; 2022 (47)**	Observational study, retrospective cohort design	335,273	The methodology involved using the EDP-Santé database, conducting multivariate logistic regression, recording variables at patient and county levels, calculating the modified Charlson Comorbidity Index, including significant variables in the model, and using R 3.6.0 software for analysis.	Admission to a stroke unit, Paralysis at the end of acute care hospitalization, Language disorder at the end of acute care hospitalization, Death at 1 year after stroke	Age, gender, modified Charlson Comorbidity Index, standard of living	Lower living standards were associated with reduced admission to stroke units, increased risk of paralysis and aphasia, and higher likelihood of death at 1 year post-stroke. Promoting access to stroke units for disadvantaged individuals is essential.	- Time between onset of symptoms and first medical contact not available<br>- Coding data for thrombolysis not reliable<br>- Unable to assess association between standard of living and thrombolysis
**Acute Ischaemic Stroke Interventions in the United States and Racial, Socioeconomic, and Geographic Disparities**<br><br>**de Havenon et al; 2021 (48)**	Retrospective, longitudinal observational study	1,439,295	The methodology involved a retrospective, longitudinal analysis of the 2016-2018 National Inpatient Sample data using multivariate logistic regression models	- Proportions of patients who received IV alteplase (tPA) and endovascular thrombectomy (EVT)	Median income in the patient's zip code, patient urban-rural location, Elixhauser Comorbidity Index, medical comorbidities (diabetes, hypertension, obesity, congestive heart failure, and intubation), sex, race/ethnicity, and age	- Utilization of tPA and EVT for IS in the United States increased from 2016 to 2018.<br>- lower SES was associated with more severe stroke<br>- Lower SES groups were less likely to have neuro-imaging (82 vs. 90%; p = 0.036) or an electrocardiogram (72 vs. 87%; p = 0.003), but differences were no longer significant on multivariate analysis.<br>- There was equal access to stroke unit care	The limitations of the study include the lack of unique patient identifiers, potential underrepresentation of cases of tPA or EVT interventions, inability to account for recurrent hospital admissions, presence of unmeasured confounding, lack of granularity in variables, and misclassification bias. Further limitations involve the absence of data on specific factors influencing IS interventions and the inability to explain the root cause of findings.
**Socioeconomic Status and Care**	Retrospective cohort study	11,050.	Patients with ischaemic stroke or transient ischaemic attack,	Rates of stroke unit admission, neurology	Use of thrombolysis, stroke unit care, neurologist	Higher income was associated with better	The limitations of the study include the use

**After Stroke Results From the Registry of the Canadian Stroke Network**<br><br>**Huang et al; 2013 (49)**			measuring SES using neighbourhood income quintiles imputed from the 2006 Canadian Census, and conducting multivariable analyses to assess differences in stroke care and medication adherence across income groups.	consultations, referrals to secondary prevention clinics, physician visits after hospital discharge, use of post discharge homecare services, and adherence to antihypertensive, antithrombotic, or lipid-lowering medications.	consultation, referrals to specialized stroke secondary prevention clinics, discharges to inpatient rehabilitation facilities, prescriptions of medications at discharge, processes of stroke care, medication adherence, age, gender.	access to certain aspects of stroke care, while individuals from lower-income areas were less likely to receive timely care and specialized services. There were no income-based differences in home care provision or medication adherence.	of income as the sole marker of socioeconomic status, the reliance on area-level income measures, the use of prescription data for medication adherence evaluation, and the potential lack of generalizability to other healthcare settings.
**Impact of areal socioeconomic status on prehospital delay of acute ischaemic stroke: retrospective cohort study from a prefecture-wide survey in Japan**<br><br>**Fukuda et al; 2023**	Retrospective observational study	9,651.	The methodology involved calculating the Areal Deprivation Index (ADI) based on census-based variables to represent the SES of different areas. The study then analysed the association of ADI with prehospital delays in both non-capital and capital areas through multivariable analyses.	- Prehospital delay defined as hospital arrival ≥4-hour after stroke onset<br>- In-hospital mortality<br>- Discharge to a nursing facility<br>- Delayed candidate arrival ≥2-hour of intravenous rt-PA<br>- Endovascular reperfusion therapy	ADI, prehospital delay, intravenous rt-PA, endovascular reperfusion therapy	Living in socioeconomically disadvantaged municipalities was associated with prehospital delays of acute ischaemic stroke in non-capital areas in Kochi prefecture, Japan. Poorer outcomes of those patients may be caused by delayed treatment of intravenous rt-PA and endovascular reperfusion therapy.	- Risk of ecological fallacy when using area-based indexes<br>- The cohort is particularly socioeconomically disadvantaged compared to the overall Scottish population
**Racial Differences in Outcomes after Acute Ischaemic Stroke Hospitalization in the United States**,<br><br>**Kumar et al; 2016 (51)**	Retrospective population-based cohort	173,910 hospitalizations representing 835,811 hospitalizations nationwide	The methodology involved using the 2011-2012 National Inpatient Sample (NIS) to identify hospitalizations with acute ischaemic stroke, conducting a retrospective population-based cohort study, and excluding cases with missing data on age, sex, and race. Data elements included diagnoses, procedures, sociodemographic data, AHRQ comorbidities, and insurance status. Comorbidities and procedures were identified using ICD-9-CM and CCS codes. Race data were collected from patients by hospital staff during registration and admission.	- In-hospital mortality<br>- Utilisation of thrombolysis<br>- Endovascular mechanical thrombectomy (EMT)<br>- Length of stay (LOS)<br>- Average inflation-adjusted charges	In-hospital mortality, utilization of thrombolysis, endovascular mechanical thrombectomy (EMT), length of stay (LOS), average inflation-adjusted charges, comorbidities, procedures	Blacks and Asians had lower in-hospital mortality rates, Blacks were less likely to receive thrombolysis and EMT, and racial minorities had higher LOS and charges compared to Whites.	- Potential for miscoding in the administrative database<br>- Survivorship bias due to stroke deaths occurring outside the hospital<br>- Unknown reasons for non-administration of thrombolysis or EMT<br>- Possibility of misclassification of race and ethnicity data<br>- Lack of a validated stroke severity measure and detailed clinical data
**Racial and Ethnic Disparities in the Utilization of Thrombectomy for Acute Stroke Analysis of Data From 2016 to 2018**	Retrospective observational study	Total: 206,853<br>- MT: 17,351	The methodology involved using the Vizient Clinical Database/Resource Manager to analyse MT utilization among different racial and ethnic groups for AIS admissions, identifying admissions through ICD-10 codes, analysing patient demographics, and conducting	- Patient sex<br>- Age<br>- Location of origin<br>- Race and ethnicity<br>- Insurance status<br>- Administration of IV-tPA	Patient sex, age, location of origin, race and ethnicity, insurance status, and administration of IV-tPA	The main findings of the study indicate that there is reduced utilization of mechanical thrombectomy in black/Hispanic patients and patients with Medicaid or uninsured status, highlighting	- Lack of information on the incidence of large vessel occlusion and stroke time of onset among different racial/ethnic groups<br>- Reliance on the accuracy of hospital billing records and ICD-10 coding

**Rinaldo et al; 2018 (52)**			multivariate linear regression analysis.			ongoing racial and ethnic disparities in access to this treatment despite its increased acceptance.	- Potential selection bias due to voluntary participation of reporting institutions<br>- Inability to analyse individual patient outcomes due to hospital-level data
**Provision of acute stroke care and associated factors in a multi-ethnic population: prospective study with the South London Stroke Register**<br><br>**Addo et al; 2021 (53)**	Observational study based on a population-based stroke register	3800	The methodology involved using data from the South London Stroke Register, multivariable logistic regression models to analyse the impact of variables on processes of care, analysis limited to complete cases	Dependent variables in this study are age, sex, ethnic origin, socioeconomic status, living circumstances before stroke, motor deficit, swallowing ability, speech impairment, visual impairments, urinary incontinence, level of consciousness, and stroke subtype.	Age, sex, ethnic origin, socioeconomic status, living circumstances, motor deficit, swallowing, speech, visual impairments, urinary incontinence, level of consciousness	Substantial increase in the provision of acute stroke care interventions over time in a multi-ethnic population.<br>Black patients compared with white patients had a significantly increased odds of admission to a stroke unit (odds ratio 1.76, 95% confidence interval 1.35 to 2.29, P<0.001) and of receipt of occupational therapy or physiotherapy (1.90, 1.21 to 2.97, P=0.01), independent of age or stroke severity.	- Missing data for some variables could potentially bias the results.<br>- The rate of missing data was generally low, but the highest rate was 12% for speech deficits.<br>- Those with missing data did not differ by age, sex, ethnicity, or level of consciousness compared to those with no missing data.
**Racial and ethnic disparities in the usage and outcomes of ischaemic stroke treatment in the United States**<br><br>**Metcalf et al; 2023 (54)**	Observational, retrospective	Total: 89,035	The methodology involved an observational analysis of ischaemic stroke patients from the 2019 National Inpatient Sample, utilising weighted logistic regressions and Poisson regression to assess the relationship between race/ethnicity and the utilization of tPA and EVT, in-hospital mortality, and length of stay. Confounding variables were controlled for, and specific diagnostic and procedural codes were used for patient classification.	- Utilization of tissue plasminogen activator (tPA)<br>- Utilization of endovascular thrombectomy (EVT)<br>- In-hospital mortality<br>- Length of stay (LOS)	- Race/ethnicity<br>- Median household income<br>- Payers<br>- Utilization of tissue plasminogen activator (tPA)<br>- Utilization of endovascular thrombectomy (EVT)<br>- In-hospital mortality<br>- Length of stay (LOS)	No Hispanic Black patients had lower odds of receiving tPA and EVT, minority populations had longer hospital length of stay after treatment, and no significant difference was found in in-hospital mortality based on race/ethnicity post-tPA or EVT. SES modified the outcomes	- Stroke severity was not considered.<br>- NIHSS is not yet coded for each patient.
**Older age, low socioeconomic status, and multiple comorbidities lower the probability of receiving inpatient rehabilitation half a year after stroke**<br><br>**Yeh et al; 2016 (58)**	The study design is a retrospective cohort study.	The participant count in this study is 488 patients with newly diagnosed stroke who received inpatient rehabilitation during the 4th to 6th months after stroke and did not die within one year.	- Retrospective cohort study using a nationally representative sample of National Health Insurance enrolees in Taiwan<br>- Included newly diagnosed stroke patients who received inpatient rehabilitation during the 4th to 6th months after stroke, excluding those who died within one year<br>- Analysed patient characteristics (age, comorbidities, etc.) and	- The probability of receiving inpatient rehabilitation during the 7th to 12th months after stroke (primary outcome)<br>- The probability of receiving inpatient rehabilitation during the 7th to 9th months after stroke (secondary outcome)<br>- The probability of receiving inpatient	- Patient characteristics: age, sex, employment status, comorbidities, catastrophic illness status, stroke type, length of stay for first admission, and other diseases<br>- Medical care provider characteristics: physician specialization, hospital accreditation level, hospital urbanization level, and hospital location<br>- Rehabilitation utilization: number of admissions in first 6	- Older age (age ≥ 75 years), low socioeconomic status, and multiple comorbidities (CCI ≥ 5) are negative predictive factors for the probability of receiving inpatient rehabilitation during the 7th to 12th months after stroke.<br>- Patients who were repeatedly admitted within the first six	- Potential inaccuracies in the use of diagnostic codes and administrative claims data<br>- Difficulty determining whether readmissions were due to recurrent stroke<br>- Lack of data on stroke severity and functional status of patients

			medical care provider characteristics as potential predictors of receiving inpatient rehabilitation during the 7th to 12th months after stroke<br>- Used generalized estimating equations (GEEs) and multivariate logistic regression to identify associated factors	rehabilitation during the 10th to 12th months after stroke (secondary outcome)	months, admission within 1 year before stroke, and inpatient/outpatient rehabilitation in different time periods (1-3 months, 4-6 months, 7-9 months, 10-12 months, 7-12 months)	months after stroke and those who received outpatient rehabilitation during the 7th to 12th months after stroke have a higher probability of receiving inpatient rehabilitation during the 7th to 12th months after stroke.<br>- The negative predictive factors (older age, low socioeconomic status, and multiple comorbidities) have a cumulative effect on the probability of receiving inpatient rehabilitation.	- Potential influence of private insurance coverage that was not accounted for in the study
**Accessibility for Rehabilitation Therapy According to Socioeconomic Status in Patients With Stroke: A Population-Based Retrospective Cohort Study**<br><br>**Yoon et al; 2023 (59)**	The study design is a population-based retrospective cohort study. It used a large nationwide database to analyse the accessibility of rehabilitation therapy for 18,842 stroke patients over a 16-year period from 2003 to 2019, based on their socioeconomic status.	The total number of participants in the study was 18,842 patients with new-onset stroke.	- Used the Korean National Health Insurance Service (NHIS) -National Sample Cohort (NSC) data, a nationwide population-based cohort<br>- Selected new-onset stroke patients based on ICD-10 codes and brain imaging evaluation<br>- Focused on "Rehabilitative developmental therapy for disorder of central nervous system (claim code: MM105)" as the main rehabilitation therapy<br>- Categorized residential area, income level, age, comorbidity, and disability severity<br>- Performed logistic regression analyses to investigate the association between socioeconomic status and rehabilitation therapy utilization	The dependent variable in this study is the utilization or receipt of continuous rehabilitation therapy, as measured by the accumulation of the "Rehabilitative developmental therapy for disorder of central nervous system (claim code: MM105)" more than 41 times.	1) Residential area (capital, urban, rural)<br>2) Income level (4 categories based on NHI premium)<br>3) Insurance type (NHI or medical aid)<br>4) Age (4 groups: <60, 60-69, 70-79, ≥80)<br>5) Comorbidity (Charlson Comorbidity Index)<br>6) Disability severity (National Disability Registration grades)<br>7) Receiving continuous rehabilitation therapy (>41 claims of MM105 code)	- Rural area and medical aid insurance type were associated with lower utilization of rehabilitation therapy after stroke.<br>- The low-middle income group showed an increased rate of receiving rehabilitation therapy compared to the lowest income group, but the middle-high- and highest-income groups did not show a significant association.<br>- The disparities in rehabilitation therapy utilization according to residential area became more significant as the duration of stroke onset increased.	- The study only focused on the MM105 claim code for rehabilitation therapy, which may have missed other important rehabilitation treatments<br>- The study used NDR grades as a proxy for stroke severity, rather than directly measuring stroke severity<br>- The study only used residential area, insurance type, and income level to represent SES, and did not include other SES indicators like education and occupation
**Socioeconomic Status and Long-Term Stroke Mortality, Recurrence and Disability in Iran: The Mashhad Stroke Incidence Study**<br><br>**Morovatdar et al; 2019 (60)**	Observational study with a 5-year follow-up period	624	The methodology involved a 5-year follow-up study using Cox regression, logistic regression, and competing risk regression models for data analysis	Mortality at 1 and 5 years post-stroke, recurrence of stroke, disability and dependency at 1 and 5 years post-stroke, severity of stroke at admission	Hyperlipidaemia, atrial fibrillation), smoking status, degree of disability after stroke, functional dependency after stroke, baseline data on age, sex, and severity of stroke based on NIHSS on admission	Unemployment prior to stroke was associated with increased post-stroke mortality, less educated patients had higher mortality rates, and individuals in less privileged areas experienced more severe strokes and higher disability rates.	- Lack of access to detailed socioeconomic variables for all residents of the neighbourhoods<br>- Inability to comment on rehabilitation plans in different zones<br>- Lack of information on medication compliance within the study period<br>- Inability to identify the most and least

							disadvantaged areas due to limited socioeconomic data<br>- Possible impact of property prices and access to healthcare facilities on study outcomes
**Association Between Socioeconomic Deprivation and Functional Impairment After Stroke The South London Stroke Register**<br><br>**Chen et al; 2015 (61)**	Observational cohort study	Total: 4414	Socioeconomic deprivation was calculated by using IMD on patient postcodes, and employing multivariate-adjusted logistic regression models to analyse the association between socioeconomic deprivation and functional impairment after stroke, with further stratification by age, sex, pre-stroke comorbidities, and stroke subtype.	Barthel index of <15	Hypertension, Myocardial infarction, Atrial fibrillation, Peripheral vascular disease, Previous transient ischaemic attack, Diabetes mellitus, Current smoking status, Barthel index before stroke, Living conditions, Glasgow Coma Scale, Urinary incontinence, Swallow impairment, Speech deficit, Motor deficit, Pathological subtypes of stroke, Hospital admission status, Stroke unit admission status, Brain imaging status, Swallow test status, Activity of daily living using the Barthel index, Health-related quality of life using the SF-12, Cognitive impairment using the mini-mental state examination or abbreviated mental test, Anxiety and depression using the hospital anxiety and depression scale	The main findings highlight a significant association between socioeconomic deprivation and functional impairment after stroke, with variations based on age, sex, stroke subtype, and pre-stroke comorbidities.	- Small sample size for patients with haemorrhagic stroke<br>- Lack of data on the National Institutes of Health (NIH) score for adjustment<br>- Failure to adjust for processes of care variables, recurrent stroke, and incident comorbidities during follow-up
**Socioeconomic Status and the Risk of Stroke Recurrence Persisting Gaps Observed in a Nationwide Swedish Study 2001 to 2012**<br><br>**Pennlert et al; 2017 (62)**	Observational study, nationwide,	Total: 168,295	Hazard regression to analyse the time to stroke recurrence while considering competing risks.	Risk of stroke recurrence, influenced by SES variables (educational level, income, living alone) and other factors like time period of first stroke, sex, age group, cardiovascular risk factors, stroke severity, stroke unit care, and type of index stroke.	Level of consciousness on admission to hospital, Hypertension, AF, diabetes mellitus, smoking status before the first stroke, Education level, Income level	Higher education and income were associated with a reduced risk of stroke recurrence, while lower educational level, low income, and living alone were linked to an increased risk of stroke recurrence, with persistent socioeconomic gaps in recurrence rates over time.	- Possible causes of bias, registration errors, and residual confounding cannot be eliminated.<br>- Total burden of stroke recurrence in Sweden during the study period was not fully assessed due to exclusion criteria.<br>- Underestimation of the true numbers of recurrent events.<br>- Underlying causes of changes in recurrence rates over time were not addressed.
**Education Level and Long-Term Mortality, Recurrent Stroke, and Cardiovascular Events in Patients With Ischaemic**	Observational study, prospective design, multi-site, non-controlled	Total: 3861.	The cumulative incidence rates of 2-year outcomes across 4 education categories were estimated using Kaplan–Meier cumulative incidence curves and compared using log-rank tests. Cox	- All-cause mortality<br>- Stroke-specific mortality<br>- Recurrent stroke<br>-Cardiovascular events	Educational level, exact years of education, demographic characteristics, socioeconomic indicators, lifestyle risk factors, medical history, medication history, baseline diastolic BP, proportions of hypertension,	Significant association between low education level and increased risk of mortality, recurrent stroke, and cardiovascular events after ischaemic stroke,	- potential selection bias<br>- residual confounding<br>- lack of data on access to stroke education

**Stroke**<br><br>**Che et al; 2020 (63)**			proportional hazards models were performed to estimate associations between education levels and outcomes within 2 years after ischaemic stroke, and hazard ratios were calculated for each stratum of education.		hyperlipidaemia, diabetes mellitus, family history of stroke, use of lipid-lowering medications, thrombotic and embolic infarcts, baseline National Institutes of Health Stroke Scale score	independently of established risk factors.	
**One-year readmission and mortality following ischaemic stroke by diabetes status, sex, and socioeconomic disadvantage: An analysis of 27,802 strokes from 2012 to 2017**<br><br>**Morton et al; 2022 (64)**	Observational prospective	Total: 25,421<br>- Diabetes: 6,857	The methodology involved identifying individuals discharged following an IS, linking data sources, stratifying outcomes by various variables, and using Cox proportional hazard models and competing risk regression for analysis, including sensitivity analyses.	1-year incidence of readmission to hospital following an IS (all-cause, cardiovascular disease, and IS), 1-year all-cause mortality rate following IS, risk of all-cause, cardiovascular, and IS readmission following IS, mortality rates following IS, readmission rates by sex, and readmission rates and mortality rates by socioeconomic disadvantage.	Diabetes status, sex, index of relative socioeconomic disadvantage (IRSD), age, readmission rates (all-cause, cardiovascular disease, IS), 1-year all-cause mortality rate	Women have higher mortality rates and IS readmission risk compared to men, and SES is associated with higher mortality rates.	- IS defined via ICD-10 codes, which may be imperfect<br>- Unable to stratify by diabetes type<br>- Limited to Victorian hospitals, potential loss to follow-up for individuals moving outside Victoria or Australia<br>- Underestimation of actual risk of IS recurrence due to only counting recurrent IS if individual was readmitted to hospital
**Impact of Poverty on Stroke Recurrence A Population-Based Study**<br><br>**Becker et al; 2024 (65)**	The study design is a population-based observational study that followed a cohort of incident stroke patients over a 3-year period to assess the relationship between neighbourhood socioeconomic status and stroke recurrence.	The total number of participants included in the analysis was 2,125 incident stroke cases.	- Population-based study using data from the Greater Cincinnati/Northern Kentucky Stroke Study (GCNKSS)<br>- Identification of all hospitalized stroke cases in the region during 2015<br>- Definition of neighbourhood socioeconomic status (nSES) based on percentage of households below the federal poverty line in each census tract<br>- Identification of recurrent stroke cases through screening of hospital admissions from 2016-2018<br>- Adjudication of recurrent stroke cases by study physicians	The dependent variable in this study is stroke recurrence.	- Age<br>- Sex<br>- Race/ethnicity<br>- Comorbidities (hypertension, diabetes, atrial fibrillation, coronary artery disease, left ventricular hypertrophy, current smoking status)<br>- Stroke type (ischemic or haemorrhagic)<br>- Neighbourhood socioeconomic status (nSES), defined by the percentage of households below the federal poverty line in each census tract	- Residents of poorer neighbourhoods had a dose-dependent increase in stroke recurrence risk, with those in the poorest neighbourhoods having more than double the risk compared to those in the richest neighbourhoods.<br>- Residents of the poorest neighbourhoods experienced recurrent strokes on average 8 years younger than those in the richest neighbourhoods.<br>- Black individuals had about twice the risk of recurrent stroke overall, and those living in the poorest neighbourhoods had nearly a fivefold increase in recurrence risk compared to White residents of the richest neighbourhoods.	- Use of neighbourhood SES rather than individual SES data<br>- Exclusion of a small number of cases due to inability to geocode addresses<br>- Inclusion of only hospitalized stroke cases, potentially missing minor strokes<br>- Limited generalizability to other racial/ethnic groups beyond the biracial population studied<br>- Potential underestimation of recurrence rates due to exclusion of events within 14 days of the index stroke
**Socioeconomic Status and the Risk of Stroke Recurrence in Chinese Patients**	Observational, longitudinal study based on a hospital-based stroke registry program.	Total: 2,294	Enrolling patients with first-ever ischaemic stroke from the Nanjing Stroke Registry Program, collecting SES information at baseline, and	Occurrence of fatal or nonfatal recurrent stroke after 7 days of the index stroke.	Disposable income and educational level	- Lower SES is associated with a higher risk of stroke recurrence in Chinese patients, with income potentially	- Single-centre study in a prosperous city, limiting generalisability

**Chen et al; 2019 (66)**			analysing the association between SES and stroke recurrence risk using a multivariate Cox regression model.			playing a more significant role than educational level.	- Lack of information on treatments and secondary preventive management<br>- Self-reported income and educational years may lead to misreporting<br>- Impact of index stroke on income not considered<br>- Some patients diagnosed without CT or MRI scans<br>- Missing information on occupation, employment status, or marital status
**Mediation Analyses of the Mechanisms by Which Socioeconomic Status, Comorbidity, Stroke Severity, and Acute Care Influence Stroke Outcome**<br><br>**Lindmark et al; 2023 (67)**	Observational study, nationwide register-based cohort study	Participant count: 25,846	Nationwide register-based cohort study including stroke using causal mediation analysis to evaluate the impact of SES on stroke outcomes and the potential effects of interventions on comorbidities, stroke severity, and acute care.	Death or ADL Dependency at 3 Months	SES (education and income), Comorbidities (diabetes, atrial fibrillation, previous stroke, prescribed drugs, smoking habits), Stroke severity (NIHSS), Acute care (reperfusion therapies, stroke unit care), Outcome: Death or ADL Dependency at 3 Months	- Low SES was associated with a higher risk of death and dependency 3 months after stroke compared to mid and high SES.<br>- Interventions targeting comorbidity, stroke severity, and acute care could potentially prevent a significant number of patients with low SES from experiencing adverse outcomes.	- The study was limited to the variables collected by the registers<br>- The method used for estimating effects may be subject to model misspecification bias.<br>- The generalizability of the study findings may be restricted to settings like Sweden, a high-income country with publicly financed education and health care systems.
**Socioeconomic Inequalities in Reperfusion Therapy for Acute Ischaemic Stroke**<br><br>**Buus et al, 2022 (68)**	Nationwide register-based cohort	37,187	Poisson regression for calculating risk ratios, selection of covariates based on directed acyclic graphs, restriction of adjusted analyses to eligible patients, handling missing data by exclusion, and the use of StataCorp 2019 for all analyses.	- Treatment with IV thrombolysis<br>- Treatment with thrombectomy	SES, treatment with IV thrombolysis or thrombectomy, age, sex, immigrant status, previous stroke, Charlson Comorbidity index score, anticoagulation therapy, hypertension, arriving hospital.	- SES influences the likelihood of receiving reperfusion therapy for ischaemic stroke, with lower SES individuals having lower treatment rates.<br>- Socioeconomic inequalities persist in reperfusion treatment rates in a country with tax-funded universal health care.<br>- Income levels and employment status show the most significant socioeconomic gradients in treatment rates.	- Lack of data on variables such as arrival with emergency medical services and ischaemic stroke subtype<br>- Possibility of coding errors in a register-based study<br>- Inclusion of some variables as broad categories may have led to residual confounding<br>- The study presumes that omitted factors had only minor overall effects

**Is there a correlation between socioeconomic disparity and functional outcome after acute ischaemic stroke?**<br><br>**Song et al; 2017 (69)**	Observational, prospective	11226	Multinomial and ordinal logistic regression models to examine associations between SES and functional outcome.	The dependent variables in the study are the different levels of disability or functional outcome measured by the modified Rankin Scale (mRS) score categories (0 to 5).	Demographics (age and gender), modified Rankin Scale (mRS), stroke risk factors (hypertension, diabetes mellitus, dyslipidaemia, cardiovascular disease, atrial fibrillation, history of stroke, smoking status, heavy alcohol consumption), admission stroke severity based on the National Institutes of Health Stroke Scale (NIHSS) score, and stroke subtype defined by the Trial of Org 10172 in Acute Stroke Treatment (TOAST) classification.	Low SES is associated with worse functional outcomes after ischaemic stroke, with a stronger impact on poor functional recovery in younger and male patients. Educational level, unemployment/low occupational class, and low income are all factors linked to worse functional outcomes.	- The CNSR dataset did not cover rural hospitals, potentially missing patients with lower SES.<br>- Lack of face-to-face follow-up by neurologists may have affected the accuracy of the mRS scores at 3 months.<br>- The data used in the study is 8-9 years old, limiting the generalisability to the current population.<br>- The study did not adjust for variables related to stroke care after discharge and stroke recurrence, which could impact the results.
**Association between socioeconomic status and post-stroke functional outcome in deprived rural southern China: a population-based stud**y<br><br>**Ouyang et al; 2018 (70)**	Observational study	425	The methodology involved door-to-door interviews, data collection using a structured questionnaire, descriptive statistics for demographic and clinical characteristics, and univariate/multivariate logistic regression to analyse the relationship between SES and functional impairment in stroke survivors.	Functional outcome of stroke survivors assessed using the modified Rankin Scale (mRS) score	Self-reported monthly family income per capita, presence of hypertension, diabetes mellitus, hyperlipidaemia, atrial fibrillation, blood pressure control, blood glucose control, smoking status, alcohol consumption, and functional outcome assessed using the modified Rankin Scale (mRS) score.	- Stroke survivors with lower income were more likely to have functional impairment, and this association remained significant even after adjusting for CVRF<br>- Poorer patients tend to have poorer post-stroke functional outcomes in deprived rural Southern China.<br>- Patients with lower SES, as indicated by average monthly family income, were at a greater risk of long-term functional impairment, even after adjusting for demographic and clinical characteristics.	- Survivor effect and recall bias<br>- A small sample size<br>- Income as a single indicator of SES may not be stable<br>- Challenges in conducting ideal population-based epidemiological stroke studies
**Association of Neighbourhood Socioeconomic Status With Outcomes in Patients Surviving Stroke**<br><br>**Stulberg et al; 2021 (71)**	Observational study, population-based stroke surveillance study	776	Data from a population-based surveillance study, calculating neighbourhood SES based on Census tract-level data, and conducting statistical analysis with adjusted generalised estimating equations.	- Functional status measured by the average of 22 activities of daily living/instrumental activities of daily living (range 1-4)<br>- Biopsychosocial health by the Stroke-Specific Quality of Life scale (range 0-5)	Functional status, Biopsychosocial health, Depressive symptoms	Higher neighbourhood SES was associated with better post-stroke outcomes, particularly in individuals with moderate to severe strokes.	- Results may not be generalizable to geographic areas with dissimilar climates<br>- This study did not account for whether individuals moved between days 0 and 90 after stroke<br>- This study does not account for post-acute

				- Depressive symptoms by the 8-item Patient Health Questionnaire (range 0-24)			care among study participants<br>- It used Census tracts as the geographic boundaries to measure neighbourhood SES<br>- There may be heterogeneity of effect among those with moderate to severe strokes<br>- Limited by potential unmeasured confounders, specifically with respect to individual-level SES
**Association between annual household income and adverse outcomes in patients who had ischaemic stroke**<br><br>**Zang et al; 2021 (72)**	Prospective cohort study, multicentre, single-blind, blinded endpoints randomized clinical trial, meta-analysis	Total: 3975	The methodology included categorising participants based on income, collecting baseline data, using logistic regression and Cox models for analysis, and conducting subgroup analyses.	Primary outcome: composite outcome of death and major disability at 3 months after stroke onset; Secondary outcomes: major disability, death, and vascular events	The measured variables in the study include annual household income per capita, demographic characteristics, lifestyle risk factors, medical history, clinical features, admission ischaemic stroke severity, ischaemic stroke subtype, blood pressure measurements, death certificates, hospital data for vascular events, and baseline characteristics of participants.	- Low annual household income per capita was significantly associated with increased risks of adverse clinical outcomes at 3 months after ischaemic stroke, independently of established risk factors.<br>- The meta-analysis confirmed the association between low annual household income per capita and adverse clinical outcomes after stroke.	- Self-reported income data may introduce bias<br>- Possibility of residual confounding
**Socioeconomic inequities in mortality and functional outcome after stroke in Zanzibar: A prospective cohort study**<br><br>**Jørgensen et al; 2023 (73)**	Prospective observational study	720	Following stroke patients for one year, assessing stroke severity and disability using scales, creating a multidimensional poverty index for measuring socioeconomic deprivation, and using Kaplan-Meier, Cox regression, and logistic regression for data analysis.	Mortality rate at 28 days and 12 months after stroke onset, functional outcome at 12 months post-stroke measured by Barthel Index (BI) and modified Rankin Scale (mRS), disability status at 12 months post-stroke, and survival time from stroke onset to death.	Demographic and socioeconomic variables, past medical history, stroke risk factors, stroke severity, age, sex, prior stroke history, level of physical activity, smoking status, presence of diabetes, functional outcome assessed by Barthel Index (BI) and modified Rankin Scale (mRS)	- High overall mortality rates at 28 days and 12 months post-stroke, with survival differences based on socioeconomic deprivation, especially significant for women.<br>- No significant difference in functional outcomes at one-year post-stroke among different SES.	- Possibility of selection bias due to non-participants who may have had more severe strokes<br>- Diagnosis of stroke based on clinical assessment in patients without neuroimaging, leading to potential misclassification
**The effects of socioeconomic and geographic factors on chronic phase long-term survival after stroke in South Korea**<br><br>**Park et al; 2022 (75)**	Retrospective longitudinal study using a population-based cohort dataset from the Korean National Health Insurance database.	6,994	Stroke patients were followed up for ten years after stroke onset.	Time to death for survival analysis, Cumulative chronic phase survival based on National Health Insurance Premium (NHIP) levels and residential areas	Age, Gender, Presence of comorbidities, Stroke subtype, Disability level (NDR grade), Residential area, Presence of university hospitals, NHIP level, Presence of ischaemic stroke	- Lower NHIP levels were associated with higher mortality risk in the chronic phase after stroke<br>- No significant independent risk factors for chronic phase mortality based on differences in residential areas	- Inability to reflect the paradigm shift with reperfusion therapy after 2015 for ischaemic stroke<br>- NHIP levels reflecting household economic status rather than individual patient economic status

						- National efforts are needed to reduce inter-regional and socioeconomic discrepancies affecting stroke management in South Korea	- Potential underrepresentation of patients with disabilities not enrolled in the NDR system<br>- Analysis limited to all-cause mortality as the endpoint without specifying the cause of death
**Impact of Socioeconomic Deprivation on Mortality in People with Haemorrhagic Stroke: a population-based cohort study**<br><br>**Mccormick et al; 2016 (76)**	Observational, longitudinal, multi-site, retrospective, stratified	Total: 782	The methodology involved using data collected between 1995 and 2011, calculating the Carstairs deprivation index, recording various risk factors, categorizing ethnic origin, classifying patients based on admission criteria, collecting follow-up data at specific intervals, and obtaining ethical approval from multiple committees.	Mortality rates of patients with haemorrhagic stroke after 1, 10, and 17 years of follow-up	Hypertension, myocardial infarction, atrial fibrillation, peripheral vascular disease, previous transient ischaemic attack, diabetes mellitus, current smoking status, accommodation type, case severity variables, pathological subtype, acute stroke care variables, socio-demographics, cardiovascular disease risk factors, stroke severity, follow-up data, Carstairs deprivation index scores, mortality rates	- The most deprived individuals had an increased risk of mortality compared to the least deprived group.<br>- The SED gradient association remained in patients with intracerebral haemorrhagic stroke, while in patients with subarachnoid haemorrhagic stroke the corresponding HRs were 2.62 (1.22-5.64), 3.03 (1.49-6.18) and 1.83 (0.87-3.83) respectively.	- Small sample size due to the low incidence of haemorrhagic stroke<br>- Lack of adjustment for processes of care, recurrent stroke, and incident co-morbidities during the follow-up period<br>- Missing data for some risk factors, affecting the ability to fully adjust for these factors<br>- Use of Carstairs deprivation index as a measure of SES without including other common socioeconomic measures such as income or education
**Socioeconomic position and cardiovascular mortality in 63 million adults from Brazil.**<br><br>**Mallinson et al; 2021 (77)**	Observational cross-sectional study using routinely collected mortality and census data	Participant count: 62.5 million	The methodology included using national census and mortality data, age-adjusted multilevel Poisson regression, defining socioeconomic position by education, focusing on adults over 20 years, and employing multiple imputation to handle missing data.	Cardiovascular mortality rate	Age, Sex, Education, Municipality of residence, Cardiovascular mortality count, Person-years at risk in 2010	Compared with women with 8+ years of education, women with <8 years of education had a three-fold risk of stroke mortality. This association was also seen for men.	The limitations of the study include potential lack of generalisability, variability in mortality reporting quality, possible misclassification of educational status, which may have influenced the outcomes.
**Association of Socioeconomic Status With Ischaemic Stroke Survival**<br><br>**Vivanco et al; 2019 (78)**	Population-based cohort study in Catalonia, Spain	Total: 16,344	Patients with first ischaemic stroke admitted to a public hospital between January 1, 2015, and December 31, 2016. Individual socioeconomic status and Primary Care Service Area Socioeconomic Index were categorized, and mixed-effects logistic and survival models were used to estimate odds ratios and hazard ratios for short-term and long-term all-cause case fatality rates.	The dependent variables in the study are short-term and long-term survival odds of death and hazard of death based on individual income levels and Primary Care Service Area (PCSA) index.	Individual socioeconomic status (categorized by income levels), Primary Care Service Area Socioeconomic Index, Cardiovascular risk factors, Reperfusion therapies	- Individual socioeconomic status was associated with short-and long-term survival in patients with ischaemic stroke.<br>- Primary Care Service Area Socioeconomic Index had only an influence on short-term survival.<br>- Interventions addressing both individuals' and primary	- Lack of data on initial stroke severity<br>- No information on patients attending private health services<br>- Possibility of different incomes within a family unit affecting the classification<br>- Intragroup differences within the ≥€18,000 income per year category

						care service socioeconomic aspects might eventually affect differently short-and long-term survival.	- Similar distribution of stroke patients in co-payment categories to the general population of Catalonia
**Sociodemographic Disparities in Long-Term Mortality Among Stroke Survivors in the United States The REGARDS Study**<br><br>**Elfassy et al; 2019 (79)**	Observational, longitudinal, retrospective, multi-site study design	1329	Long-term mortality was defined as the time from 30 days post-stroke to date of death or censoring, and using mortality rate ratios to compare mortality rates by demographic and socioeconomic factors.	Long-term mortality among stroke survivors	Age, Sex, Race, Educational attainment, Marital status, Household income, Health insurance status, Geographical location, Urban/rural/mixed environment, Neighbourhood socioeconomic status	Older individuals and men had higher long-term mortality rates among stroke survivors. Lower educational attainment, lower income, and lower neighbourhood SES were associated with increased long-term mortality. There were no differences in long-term mortality rates by race, rurality, or US region.	Neighbour SES did not account for differences across the US or by geographic period
**Neighbourhood Differences in Post-Stroke Mortality**<br><br>**Osypuk et al; 2017 (81)**	Prospective cohort study, observational design	Total: 15,560<br>Total with multiple imputation: 17,960	Prospective cohort data from the Health and Retirement Study (HRS) with participants born between 1900 and 1947. Exclusions were made based on specific criteria, resulting in a final analytic sample of 15,560 participants. Biennial interviews were conducted through 2010 with retention rates over 80%.	Mortality rates and risks associated with stroke survivors and individuals without a history of stroke	Neighbourhood SEPS variables (average tract family income, percent of residents below the poverty line, index of deprivation) and individual-level variables (demographic, socioeconomic, behavioural risk factors, health conditions)	Neighbourhood factors like disadvantage, racial composition, and social ties predict survival of stroke patients, with effects similar for stroke survivors and individuals without a history of stroke, indicating broader mortality risk factors beyond stroke care.	- Uncertainty in causality due to potential confounders or factors on the causal chain not being adjusted for<br>- Less statistical power in models examining time since stroke<br>- Potential biases in the study design and analysis<br>- Lack of information on stroke severity and subtypes<br>- Uncertainty in generalising the findings to younger populations or different geographic locations
**Socioeconomic status and stroke severity: Understanding indirect effects via risk factors and stroke prevention using innovative statistical methods for mediation analysis**<br><br>**Lindmark et al; 2022 (83)**	Observational retrospective register-based cohort study	Total: 86,316	The methodology includes retrieval of SES data, use of education level as a proxy for SES, mediation analysis for pathways via risk factors and stroke prevention drugs, causal inference approach to mediation, and estimation of effects through Monte Carlo simulation.	Stroke severity	Education level, Stroke severity	- Low education was associated with a higher risk of severe stroke compared to mid/high education, with almost one-third of this association explained by risk factors.<br>- The study suggests that addressing risk factors more aggressively could help decrease stroke severity in individuals with low education levels.	The limitations of the study include the use of complete case analysis due to the computationally intensive estimation method, potential residual confounding in observational studies, assumptions about unobserved confounders, restrictions imposed by the predefined set of variables collected in

						the register, potential underestimation of the mediating role of risk factors, the need for higher data resolution to explore more factors, and the limited generalisability of the findings to countries with different demographics and healthcare systems.
